# Targeting microglial MyD88 reprograms injury-activated microglia and promotes spinal cord repair via TGF-β signaling and biomimetic nanodelivery

**DOI:** 10.7150/thno.136733

**Published:** 2026-07-13

**Authors:** Jintao Liu, Beibei Yu, Wanjun Cao, Jilu Liu, Yundi Gao, Honghui Mao, Xiaochuan Gao, Lingli Guo, Shengyou Li, Qiao Huang, Mingze Qin, Fang Kuang, Jinghui Huang

**Affiliations:** 1 Department of Orthopedics, Xijing Hospital, Fourth Military Medical University, Xi'an 710032, China.; 2 Department of Neurobiology, School of Basic Medicine, Fourth Military Medical University, Xi'an 710032, China.; 3 The Shaanxi Province Key Laboratory of Brain Function Analysis and Modulation, Xi'an 710032, China.; 4 Department of Orthopedics, Naval Hospital of Eastern Theater, Zhoushan 316000, China.; 5 Department of Neurosurgery, The First Affiliated Hospital of Chongqing Medical University, Chongqing 400016, China.; 6 Seventh Squad, Second Regiment, Basic Medical College, Fourth Military Medical University, Xi'an 710032, China.

**Keywords:** MyD88, microglial state transition, spinal cord injury, TGF-β, biomimetic nanoparticle

## Abstract

**Background:**

Following spinal cord injury (SCI), activated microglia sustain neuroinflammation and drive secondary tissue damage, and this limits functional recovery. Myeloid differentiation primary response 88 (MyD88) is a central adaptor of innate immune signaling, but whether and how microglial MyD88 regulates state transition after SCI remains unclear. This study aims to explore the impact of microglial MyD88 signaling on microglial state trajectories and tissue repair after SCI and to develop a microglia-targeted delivery strategy for therapeutic modulation.

**Methods:**

A forceps-mediated SCI mouse model was established, and bulk and single-cell RNA sequencing were used to profile the temporal activation of microglial MyD88 signaling in the spinal cord. Pharmacological MyD88 inhibition (ST2825) and inducible microglia-specific MyD88 conditional knockout mice (*Tmem119*^CreERT2^;*Myd88*^fl/fl^) were used to assess neuroinflammation, tissue remodeling and downstream signaling. Functional recovery was evaluated by behavioral testing, bidirectional axonal tracing and electrophysiology. For translational validation, microglia membrane-coated, peptide-modified biomimetic nanoparticles (ST2825·DSPE@MG) were engineered to enhance microglia targeting and therapeutic efficacy.

**Results:**

Transcriptomic analyses revealed rapid and sustained activation of MyD88 signaling preferentially in microglia after SCI. ST2825 suppressed pro-inflammatory outputs *in vitro* and *in vivo* while preserving trophic mediators. Microglia-specific MyD88 deletion reprogrammed injury-activated microglia from a pro-inflammatory state to a repair-associated phenotype, reducing neuronal damage, preserving axons and improving locomotor recovery. TGF-β receptor blockade with LY2109761 abolished the protective effects of MyD88 deficiency. Additionally, ST2825·DSPE@MG nanoparticles exhibited microglia-targeted uptake and conferred superior therapeutic efficacy.

**Conclusion:**

Our data establish MyD88 as a critical regulator of microglial reprogramming after SCI and highlight its potential as a therapeutic target for spinal cord repair.

## Introduction

Spinal cord injury (SCI) initiates an immediate mechanical insult followed by a prolonged secondary injury cascade 1. This secondary phase, characterized by sustained neuroinflammation, oxidative stress and scar formation, contributes to long-term neurological deficits and limits endogenous repair capacity 2, 3. Despite advances in acute management, effective strategies that selectively modulate secondary injury processes remain limited, highlighting the need to define actionable cellular and molecular regulators of the post-injury microenvironment 4, 5.

Resident microglia are central to this inflammatory response 6. As the principal innate immune cells of the central nervous system (CNS), microglia rapidly sense injury-associated danger signals and undergo extensive transcriptional and functional remodeling 7, 8. Rather than adopting binary phenotypes, injury-activated microglia exist along a dynamic spectrum of states that can either exacerbate secondary injury or facilitate repair processes, including debris clearance, trophic signaling and tissue remodeling 9. Single-cell transcriptomic studies have further highlighted marked heterogeneity and plasticity of microglia responses after SCI 10-12. However, the signaling nodes that govern state transitions, their temporal coordination, and their linkage to repair-relevant outcomes are still poorly understood 13. This gap constrains the development of state-dependent immunomodulatory interventions.

Myeloid differentiation primary response 88 (MyD88) is a core adaptor of innate immune signaling 14, 15. Downstream of most Toll-like receptors (TLRs), MyD88 transduces damage-associated molecular pattern (DAMP) cues to NF-κB pathways, driving cytokine and chemokine production 16, 17. Although our previous research demonstrated that global inhibition of MyD88 signaling could reduce neuroinflammation after CNS injury, such approaches lack cellular specificity and temporal precision, potentially suppressing protective immune functions while masking cell-specific mechanisms 18. The microglia-intrinsic contribution of MyD88 across distinct phases of SCI has not been systematically examined. Furthermore, it is unclear whether manipulating MyD88 can actively redirect microglial state trajectories toward sustained repair-associated programs, rather than simply dampening inflammatory output.

In this study, we integrated bulk and single-cell transcriptomics with pharmacological inhibition, inducible microglia-specific genetic manipulation and translational nanomedicine engineering to suppress microglial MyD88 signaling after SCI. Our data showed that MyD88 signaling was rapidly induced and persistently engaged in microglia following injury and was associated with pro-inflammatory microglial programs and delayed recovery of homeostasis. Targeted MyD88 suppression reprogrammed microglia from an inflammatory to a migrating state and improved tissue preservation, reduced scarring, enhanced connectivity and promoted locomotor recovery. We further identified TGF-β signaling as required for the protective phenotype. Finally, we developed a microglia-targeted biomimetic nanoparticle platform to deliver the MyD88 inhibitor ST2825, which provided therapeutic benefits, establishing a proof-of-concept strategy for precision immunomodulation after SCI.

## Materials and Methods

### Animals

Adult female mice (8-10 w, 20-25 g) were obtained from the Experimental Animal Center of Air Force Medical University (Xi'an, China). To achieve microglia-specific deletion of *Myd88*, *Tmem119*^CreERT2^ mice (The Jackson Laboratory, stock no. 031820) were crossed with *Myd88*^fl/fl^ mice (stock no. 009108), generating *Tmem119*^CreERT2^;*Myd88*^fl/fl^ conditional knockout (cKO) mice 19. *Myd88*^fl/fl^ littermates served as wild-type (WT) controls. Animals were housed under specific pathogen-free conditions (12 h light/dark cycle) with ad libitum access to food and water. Investigators were blinded to genotype and treatment during data acquisition and quantification when feasible.

### Tamoxifen-induced recombination

Tamoxifen (TAM; MedChemExpress, HY-13757A) was dissolved in corn oil and administered by oral gavage at 200 mg/kg once daily from day -11 to day -7 relative to SCI. SCI was performed 7 days after the final TAM administration. Microglia-restricted recombination was validated in uninjured spinal cord sections using immunofluorescence staining for TMEM119 and MyD88.

### SCI model

A forceps-mediated spinal cord crush injury model was used 20. Mice were anesthetized with isoflurane (1.5-2.5% in oxygen), placed on a heating pad, and subjected to laminectomy at the T9 vertebral level. A No. 5 Dumont forceps (Fine Science Tools) fixed on a stereotaxic apparatus was applied to the exposed spinal cord for 5 s to induce a standardized crush injury. Sham-operated mice underwent laminectomy without forceps application. Postoperative care included buprenorphine for analgesia twice daily for 3 days and manual bladder expression twice daily until recovery.

### Experimental design and tissue collection

For temporal profiling of MyD88 signaling, lesion-centered spinal cord tissue was collected at 9, 18, 36 and 72 h post-injury for immunoblotting, and at 3, 7, 14 and 28 days post-injury (dpi) for immunofluorescence analyses; for bulk RNA sequencing (RNA-seq) and single-cell RNA sequencing (scRNA-seq), a 1 cm spinal cord segment centered on the lesion epicenter (extending 0.5 cm rostral and 0.5 cm caudal) was collected at 3 dpi; for histological analyses, tissues were collected at 3 dpi (neuronal preservation) and 28 dpi (scar and axonal markers). Bidirectional axonal tracing was performed at 10-13 weeks post-injury. Sample sizes (n) for each experiment are detailed in the corresponding figure legends.

Exclusion criteria included perioperative death, incomplete injury, severe surgical complications, and marked body weight loss beyond predefined thresholds (≥ 20%).

### *In vivo* pharmacological inhibition of MyD88 signaling (ST2825)

ST2825 (MedChemExpress, HY-50937) was dissolved in PBS containing 0.1% DMSO and administered immediately after injury by bilateral intraspinal microinjection into the lesion site at a total dose of 2.5 µg per mouse (1.25 µg/side in 0.5 µL; total injection volume 1.0 µL) according to an established dosing strategy 21, 22. Injections were performed with a Hamilton syringe fitted with a glass micropipette at 50 nL/min (0.5 µL per site) at 0.3 mm lateral and 0.7 mm depth from the dorsal surface. Vehicle-injected mice served as controls.

### TGF-β receptor blockade (LY2109761)

To test the requirement of TGF-β signaling, LY2109761 (MedChemExpress, HY-12075) was administered by oral gavage at 50 mg/kg twice daily for 3 consecutive days starting immediately after SCI 23. Corn oil was used as the vehicle control. TGF-β pathway activity was assessed by immunofluorescence for nuclear phosphorylated SMAD3 (pSMAD3) at 3 dpi.

### Cell culture experiments

The N9 murine microglial cell line was cultured in DMEM supplemented with 10% fetal bovine serum and 1% penicillin/streptomycin at 37 °C in 5% CO₂. Cells were pretreated with control peptide (CP) or MyD88 inhibitory peptide (MIP; Novus Biologicals, NBP2-29328) at 100 µM for 24 h, followed by stimulation with LPS (Sigma, L2630, 100 ng/mL) or IL-4 (MedChemExpress, HY-P70653, 40 ng/mL) for 24 h 24.

### Primary microglia culture and ST2825 treatment

Primary microglia were prepared from P0-P1 neonatal mice as previously described with minor modifications. Briefly, cerebral cortices were dissociated with trypsin and plated to generate mixed glial cultures. After 10 days, microglia were isolated by shaking and seeded on poly L-lysine-coated coverslips at 1 × 10^5^ cells/cm². Cells were pretreated with ST2825 (5 µM) or vehicle for 12 h, followed by LPS stimulation (100 ng/mL) for 12 h. Supernatants were collected for multiplex ELISA, and cells were fixed for immunofluorescence staining.

### Multiplex ELISA

TNF-α, IL-1β, IL-10, CSF-1, TGF-β1 and IGF-1 in culture supernatants were quantified with multiplex ELISA kits (Ruixin Biotech; RX202412M, RXW203063M, RX203075M, RXW200222M, RXW202402M, RXW202483M) according to the manufacturer’s instructions. Concentrations were calculated from standard curves.

### Immunofluorescence staining and image acquisition

Mice were perfused transcardially with PBS followed by 4% paraformaldehyde (PFA). Spinal cords were post-fixed in 4% PFA for 4 h, cryoprotected in 30% sucrose, embedded and sectioned longitudinally at 14 µm. Sections were permeabilized and blocked in 5% normal donkey serum containing 0.3% Triton X-100 and incubated with primary antibodies against MyD88, Iba1, F4/80, GFAP, iNOS, Arg-1, TMEM119, pSMAD3, NF200, GAP43, ChAT, TUJ1 and synaptophysin (Table S1). After incubation with fluorophore-conjugated secondary antibodies, nuclei were counterstained with DAPI.

Images were acquired with an Olympus FV3000 laser-scanning confocal microscope or an Olympus VS200 slide scanner under identical acquisition settings. Quantification was done using ImageJ in predefined regions of interest (ROIs) spanning the lesional and peri-lesional regions. For high-magnification views, confocal Z-stacks were taken with a step size of 0.5-1 µm over a total depth of 5-10 µm. Maximum intensity projections were generated in Olympus FV31S-SW software for subsequent quantification.

### Histology and scar quantification

For Nissl staining, sections were processed using cresyl violet and imaged using an Olympus VS200 microscope. For peri-lesional neuron quantification, three standardized ROIs (200 × 200 µm each) were randomly selected in the gray matter 300 µm rostral and caudal to the lesion border. Nissl-positive neurons were identified by the following criteria: cell diameter > 15 µm, presence of a clear nucleolus, and abundant cytoplasmic Nissl substance with a tigroid appearance. Data for each animal represent the mean of three ROIs. For hematoxylin and eosin (H-E) staining, sections followed standard protocols, and scar area was quantified at 28 dpi. Scar area was considered as the non-neural dense tissue region and cavitation within the lesion boundary, and ImageJ was used for quantification.

### Western blotting

Lesion-containing spinal cord tissues were homogenized in RIPA buffer supplemented with protease and phosphatase inhibitors. Protein concentration was determined using a BCA assay. Equal amounts of protein were separated by SDS-PAGE, transferred to PVDF membranes and probed with antibodies against TLR4, MyD88, TNF-α, IL-1β, iNOS, Arg-1, GAP43 and β-actin (Table S1). Signals were detected using enhanced chemiluminescence and quantified by densitometry (ImageJ). Target band intensity was normalized to β-actin.

### Bulk RNA-seq and bioinformatics

For bulk RNA-seq, each sample was derived from one individual mouse, and no biological pooling was performed. Thus, each group included 5 independent biological samples (n = 5 mice per group). Sequencing was performed on an Illumina NovaSeq 6000 (PE150 mode) following cluster generation via the cBot system. After quality control (removal of adapters, low-quality reads and duplicates), clean reads were mapped to the mm10 reference genome using Hisat2. Gene abundances were normalized to TPM. Differential expression analysis was executed with limma (v3.52.1), applying thresholds of *p* < 0.05 and ∣log_2_FC∣ > 0.575. Functional annotation, including GO and GSEA, was performed using clusterProfiler (v4.9.3). Visualization was generated via GseaVis (v0.0.9) and ggplot2 (v3.3.6) 25, 26.

### scRNA-seq and analyses

Single-cell suspensions were prepared from pooled spinal cord tissue (5 mice per sample) centered on the lesion epicenter at 3 dpi, and scRNA-seq libraries were constructed with the GEXSCOPE® Single-Cell RNA Library Kit (Singleron Biotechnologies) according to the manufacturer's instructions. Libraries were sequenced on the Illumina NovaSeq 6000 platform. Raw sequencing reads were processed through the Singleron analysis pipeline, including read alignment, cell barcode/UMI processing and generation of the gene–cell expression matrix. After removing doublets with DoubletFinder (v2.0.3), we retained high-quality cells with 300-10,000 features, < 20% mitochondrial genes, and < 0.1% hemoglobin genes. After UMAP clustering in Seurat (v4.4.0), cell identities were assigned with SingleR and canonical marker genes. Pseudotime trajectories were reconstructed by Monocle2 (v2.22.0). Gene set activity scoring relied on AUCell (v1.18.1) to quantify pathway signatures 27. In addition, we used the Tabulae Paralytica single-cell atlas 28 to independently assess the expression of *Myd88* across major cell types after SCI.

### Behavioral testing

Hindlimb locomotor function was assessed using the Basso Mouse Scale (BMS) 29 by two independent observers blinded to group allocation. CatWalk XT gait analysis (Noldus) 30 was performed at 28 dpi to quantify average speed, stride length, initial dual stance and terminal dual stance. Mice were acclimated to the apparatus for 3 days. Runs were included only if they met predefined criteria (uninterrupted run; speed variation < 60%).

### Axonal tracing

Bidirectional tracing was performed at 10 weeks post-injury 31. For anterograde tracing, rAAV9-hSyn-EGFP (2.00 × 10^12^ vg/mL; BrainCase) was injected cranially to the lesion at four sites (1 µL/site, 50 nL/min). For retrograde tracing, Fluoro-Gold (4% in H₂O; Santa Cruz, sc-358883) was injected caudally to the lesion (100 nL, 50 nL/min). Spinal cords were harvested at 10 weeks + 5 days (Fluoro-Gold) or 13 weeks (EGFP) post-injury as indicated. EGFP and Fluoro-Gold signals were quantified in standardized ROIs cranial and caudal to the lesion border.

### Compound muscle action potential (CMAP) recording and analysis

CMAP recordings were performed at 10 weeks post-injury. Briefly, a bipolar stimulating electrode was placed on the exposed spinal cord cranial to the lesion site, and a recording electrode was inserted into the hindlimb gastrocnemius muscle. Latency was measured from the stimulus artifact onset to the first deflection of the CMAP waveform from baseline. Amplitude was measured as the peak-to-peak voltage. All recordings were conducted under constant body temperature (37 °C) with standardized electrode placement.

### Biomimetic nanoparticle fabrication and characterization

#### MG1-modified fluorescent liposomes (vehicle)

SPC, cholesterol, DSPE-PEG-MG1 (MG1 sequence: CHHSSSARC, cyclized via Cys1-Cys9) and DiD (all from Shanghai Apeptide Co.,Ltd) were co-dissolved in 1 mL chloroform. Solvent was removed under reduced pressure to form a thin lipid film, which was hydrated with ultrapure water under sonication. The suspension was processed by bath sonication (180 W) and extruded using a mini-extruder (Avanti) through a 100-nm polycarbonate membrane (Whatman) to obtain MG1-modified fluorescent liposomes.

#### Microglia membrane-coated, MG1-modified, ST2825-loaded liposomes

SPC, cholesterol, DSPE-PEG-MG1, DiD and ST2825 were co-dissolved in 1 mL of chloroform and processed by thin-film hydration, bath sonication (180 W), and extrusion through 100-nm polycarbonate membranes to yield ST2825-loaded MG1-modified liposomes.

Liposomes were then mixed with primary microglia membranes at a 1:1 mass ratio, sonicated in an ice bath for 30 s, and incubated at room temperature for 2 h to generate MG1-modified, microglia membrane-coated, DiD-labeled vehicles or ST2825-loaded nanoparticles (DSPE@MG or ST2825·DSPE@MG). Drug loading and encapsulation efficiency were 6.2% and 93.8%, respectively.

### *In vivo* lesion retention and cellular uptake

For lesion targeting, DiD-labeled nanoparticles were administered locally after SCI. At 3 days after administration, spinal cords were harvested for *ex vivo* fluorescence imaging using an IVIS Spectrum system (PerkinElmer) and radiant efficiency profiling along the rostrocaudal axis. Spinal cord sections were immunostained with Iba1 to detect nanoparticle colocalization with microglia.

### Statistical analysis

We conducted statistical analyses using R (v4.2.0) and GraphPad Prism (v8.0.2). All data are shown as mean ± SD. Normality was assessed with the Shapiro-Wilk test; appropriate data transformations or nonparametric tests were applied when necessary. Missing data were not imputed unless specified. For two-group comparisons, an unpaired two-tailed Student’s *t*-test was used for normally distributed data, and the Wilcoxon rank-sum test was applied for non-normal data. For comparisons among multiple groups, one-way ANOVA or two-way repeated-measures ANOVA followed by Tukey’s post hoc test was applied for normally distributed data; otherwise, the Kruskal-Wallis test with Dunn’s post hoc test was used for non-normally distributed data. We used Spearman’s rank correlation for correlation analyses. *p* < 0.05 was considered statistically significant. All experiments included at least three biological replicates unless stated otherwise in the figure legends. All comparisons across multiple related readouts were viewed as exploratory analyses; accordingly, no formal correction for multiplicity was applied.

## Results

### Spatiotemporal activation of MyD88 signaling in microglia after SCI and association with microglial state markers

**Figure 1:**
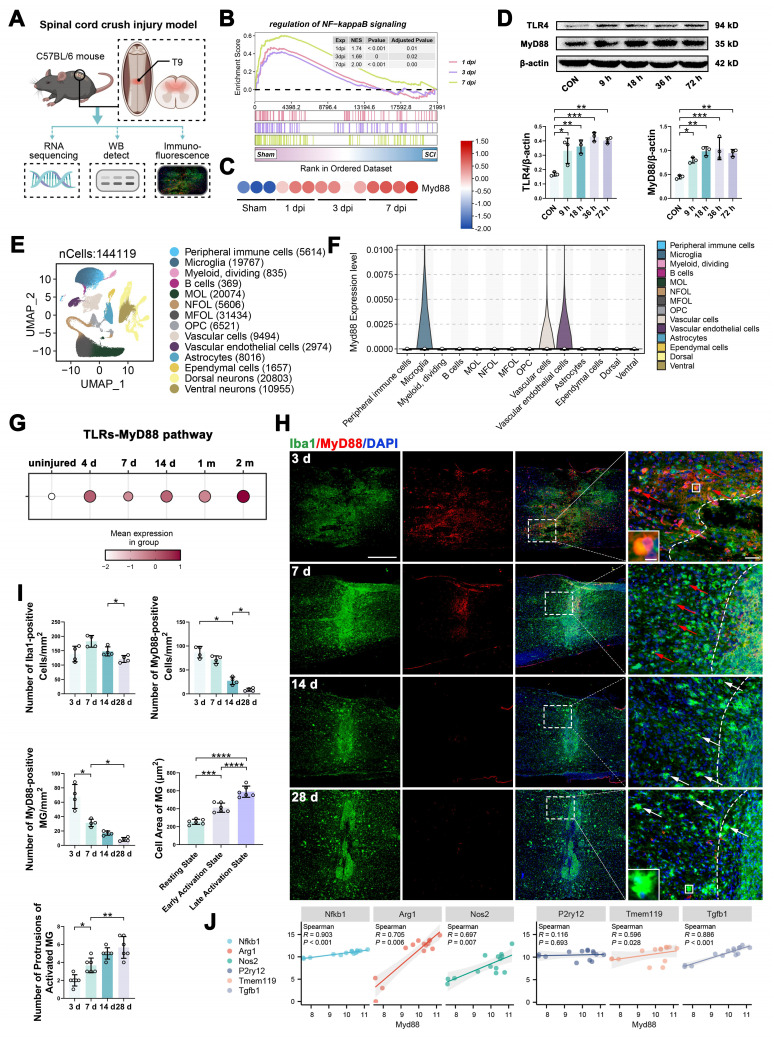
MyD88 signaling is rapidly induced and predominantly engaged in microglia after SCI. (A) Schematic of the forceps-mediated spinal cord crush model and workflow for RNA-seq, immunoblotting, and immunofluorescence. (B) GSEA showing enrichment of the "regulation of NF-κB signaling" gene set at 1, 3, and 7 dpi. (C) *Myd88* expression ranking in bulk RNA-seq datasets from sham and SCI spinal cord tissues at 1, 3, and 7 dpi. (D) Representative immunoblots and densitometric quantification of TLR4 and MyD88 in injured spinal cord tissue across the acute phase; normalized to β-actin. Data are mean ± SD; n = 3 mice/group; one-way ANOVA with Tukey’s post hoc test. (E) UMAP of cells from the Tabulae Paralytica SCI single-cell atlas colored by *Myd88* expression. (F) Violin plots of *Myd88* expression across annotated cell populations. (G) AUCell scoring of TLR-MyD88 pathway activity in microglia over time. (H) Representative longitudinal sections at 3, 7, 14, and 28 dpi stained for MyD88 (red) and Iba1 (green); DAPI (blue). Dashed lines indicate lesion boundaries; boxed regions show higher magnification. (I) Quantification of Iba1^+^ cell density, MyD88^+^Iba1^+^ microglial density, soma area, and protrusion number at 3, 7, 14, and 28 dpi. Data are mean ± SD; n = 4 mice/group; one-way ANOVA or two-way repeated-measures ANOVA followed by Tukey’s post hoc test. MG: microglia. (J) Spearman correlations of expression level of mRNA FPKM between *Myd88* and microglial marker genes (*P2ry12*,* Tmem119*,* Nos2*,* Nfkb1*,* Arg1*,* Tgfb1*) during the acute stage. Correlation analysis was based on bulk RNA-seq data from lesion-centered spinal cord tissues at 1, 3, and 7 dpi (n = 4, 5, and 5 independent samples per time point, respectively). Scale bars: 500 μm (overview), 50 μm (insets), 10 μm (single-cell views). Statistical significance: *p < 0.05, **p < 0.01, ***p < 0.001, ****p < 0.0001; ns, not significant.

To examine the temporal dynamics of MyD88 signaling following SCI, we used a forceps-mediated crush injury model (Figure 1A). We reanalyzed our previously generated bulk RNA-seq dataset 32 by gene set enrichment analysis (GSEA) and found notable enrichment of NF-κB signaling downstream of MyD88 at 1, 3, 7 dpi (Figure 1B). Consistently, *Myd88* mRNA abundance at the lesion site increased across these time points relative to that in the sham controls (Figure 1C). At the protein level, immunoblotting demonstrated that TLR4 and MyD88 were upregulated as early as 9 h post-injury and remained elevated for 72 h, indicating rapid and sustained activation of the TLR-MyD88 axis during the acute phase (Figure 1D).

To localize *Myd88* expression across spinal cord cell types, we reanalyzed the Tabulae Paralytica SCI single-cell atlas 28. Among 14 annotated populations, *Myd88* expression was highest in microglia and vascular endothelial cells (Figure 1E-F). Within microglia, AUCell-based pathway scoring indicated sustained activity of the TLR-MyD88 gene program across the post-injury time course (uninjured to 2 months post-injury) (Figure 1G). This pointed to microglia as the primary spinal cell type exhibiting sustained MyD88-dependent signaling after SCI.

We next validated these observations in our SCI model by tracking immunofluorescence changes over time at 3, 7, 14 and 28 dpi. MyD88 immunoreactivity rose sharply at 3 and 7 dpi and predominantly colocalized with Iba1^+^ microglia at the lesion site (Figure 1H). Quantification revealed that the density of MyD88^+^Iba1^+^ microglia peaked at 3 dpi and progressively declined thereafter (Figure 1I). In parallel, microglia exhibited robust activation early after injury and subsequently underwent morphological remodeling, including an increased soma area and higher protrusion numbers at later stages (Figure 1I). Finally, to relate *Myd88* expression to microglial state markers during the acute stage, we assessed correlations between *Myd88* and canonical marker genes at 1, 3, 7 dpi. *Myd88* did not correlate with the homeostatic marker *P2ry12* but showed significant positive correlations with *Nfkb1*, *Nos2*, *Arg1* and *Tgfb1* (all *p* < 0.01) (Figure 1J), indicating that elevated *Myd88* is associated with activated microglial state remodeling rather than with baseline homeostasis.

### MyD88 inhibition induces phenotypic and secretory reprogramming of microglia *in vitro* and *in vivo*

To ask whether suppression of MyD88 signaling can shift microglial response programs, we began with the N9 microglial cell line. We pretreated cells with a control peptide (CP) or a MyD88 inhibitory peptide (MIP; Novus Biologicals, NBP2-29328) and then stimulated them with LPS or IL-4 to bias pro-inflammatory or repair-associated programs, respectively. MIP pretreatment reduced LPS-induced iNOS immunoreactivity compared to CP-treated controls (Figure S1A). Additionally, Arg-1^+^ cells were detected under MIP-treated conditions (MIP-CON and MIP-LPS) and displayed a more ramified morphology resembling that of the IL-4-positive control (Figure S1A-B), indicating that blocking MyD88 reduces LPS-induced inflammation while promoting repair *in vitro*.

**Figure 2:**
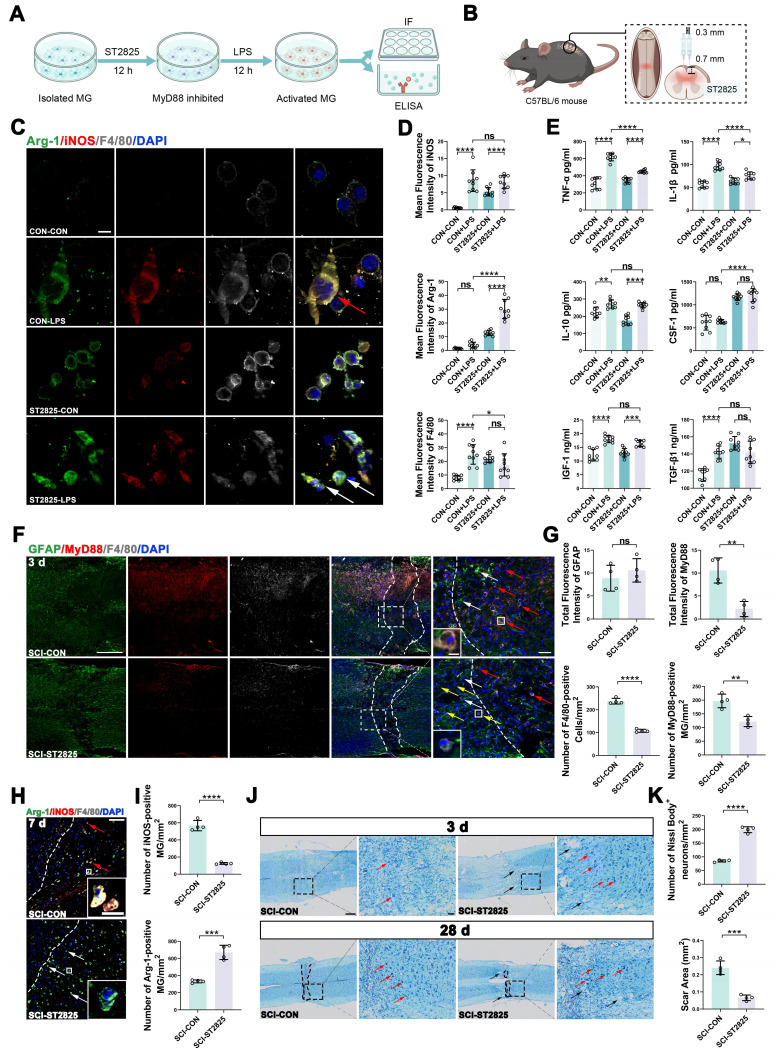
ST2825 reprograms microglial inflammatory output *in vitro* and *in vivo*. (A) *In vitro* workflow for primary microglia: ST2825 pretreatment followed by LPS stimulation. (B) *In vivo* design: bilateral intraspinal ST2825 injection immediately after SCI. (C) Representative primary microglia stained for Arg-1 (green), iNOS (red), F4/80 (white), and DAPI (blue). Scale bar: 10 μm. (D) Quantification of iNOS, Arg-1, and F4/80 mean fluorescence intensity in primary microglia. Data are mean ± SD; one-way ANOVA with Tukey’s post hoc test. (E) Multiplex ELISA quantification of secreted TNF-α, IL-1β, IL-10, CSF-1, TGF-β1, and IGF-1 levels. Data are mean ± SD; one-way ANOVA with Tukey’s post hoc test. (F) Representative spinal cord sections at 3 dpi stained for GFAP (green), MyD88 (red), F4/80 (white), and DAPI (blue). (G) Quantification of GFAP, MyD88 fluorescence intensity, densities of F4/80^+^ cells and MyD88^+^ microglia at 3 dpi. Data are mean ± SD; n = 4 mice/group; unpaired two-tailed *t*-test. (H) Representative sections at 7 dpi stained for Arg-1 (green), iNOS (red), F4/80 (white), and DAPI (blue). (I) Quantification of iNOS^+^ and Arg-1^+^ microglia at 7 dpi. Data are mean ± SD; n = 4 mice/group; unpaired two-tailed *t*-test. (J) Representative Nissl staining at 3 and 28 dpi. Scale bars: 400 μm (overview) and 100 μm (enlarged). (K) Quantification of Nissl body-positive neurons at 3 dpi and scar area at 28 dpi. Data are mean ± SD; n = 4 mice/group; unpaired two-tailed *t*-test. Scale bars: 500 μm (overview), 50 μm (insets), 10 μm (single-cell views) unless otherwise indicated. Statistical significance: *p < 0.05, **p < 0.01, ***p < 0.001, ****p < 0.0001; ns, not significant.

We next validated these findings in primary microglia with a different MyD88 inhibitor, ST2825 (Figure 2A). CCK-8 assays confirmed that ST2825 at 5 µM for 12 h did not cause significant microglial cytotoxicity or apoptosis (Figure S1C-D). In vehicle-treated cultures, LPS induced amoeboid morphology and robust iNOS expression (CON-LPS; Figure 2C-D). By contrast, ST2825 pretreatment reduced iNOS signal and increased Arg-1 immunoreactivity in LPS-stimulated microglia (ST2825-LPS; Figure 2C-D). Consistent with this, ST2825-treated microglia exhibited smaller somata with elongated bipolar processes, generating rod-like morphologies (Figure 2C). To assess functional output, cytokines and growth factors in the culture supernatants were measured by multiplex ELISA. ST2825 markedly reduced LPS-induced TNF-α and IL-1β levels, whereas IL-10, CSF-1, IGF-1 and TGF-β1 levels were preserved compared to those in the vehicle-LPS controls (Figure 2E). CSF-1, TGF-β1 and IGF-1 were detectable at relatively high levels under resting conditions (CON-CON and ST2825-CON) (Figure 2E). These data suggest that MyD88 inhibition dampens pro-inflammatory outputs while maintaining repair-associated secretions in primary microglia.

We then explored whether ST2825 modulated microglia responses *in vivo* after SCI (Figure 2B). ST2825 was bilaterally injected into the lesion site immediately after injury. At 3 dpi, MyD88 immunoreactivity at and around the lesion epicenter was reduced in ST2825-treated mice compared to that in vehicle controls (Figure 2F-G), accompanied by fewer F4/80^+^ cells and a reduced density of MyD88^+^ microglia within the lesion (Figure 2G). In the same regions, GFAP signal intensity did not differ between groups (Figure 2F-G), suggesting preferential effects on microglia at an early time point. At 7 dpi, vehicle-treated mice showed abundant iNOS^+^ microglia in peri-lesional areas, whereas ST2825 reduced iNOS^+^ microglia and increased Arg-1^+^ microglia (Figure 2H-I), consistent with an *in vivo* shift in marker profiles. To evaluate the tissue-level consequences, we performed Nissl staining. At 3 dpi, ST2825-treated mice exhibited higher densities of Nissl body-positive neurons in peri-lesional regions (Figure 2J-K). At 28 dpi, scar area was reduced in the ST2825 group, although tissue cavitation at the injury epicenter remained evident (Figure 2J-K). Collectively, these findings link acute MyD88 inhibition to microglial reprogramming and neuronal preservation, with sustained effects on tissue remodeling.

### Microglia-specific MyD88 deletion attenuates neuroinflammation and accelerates recovery of homeostasis after SCI

**Figure 3:**
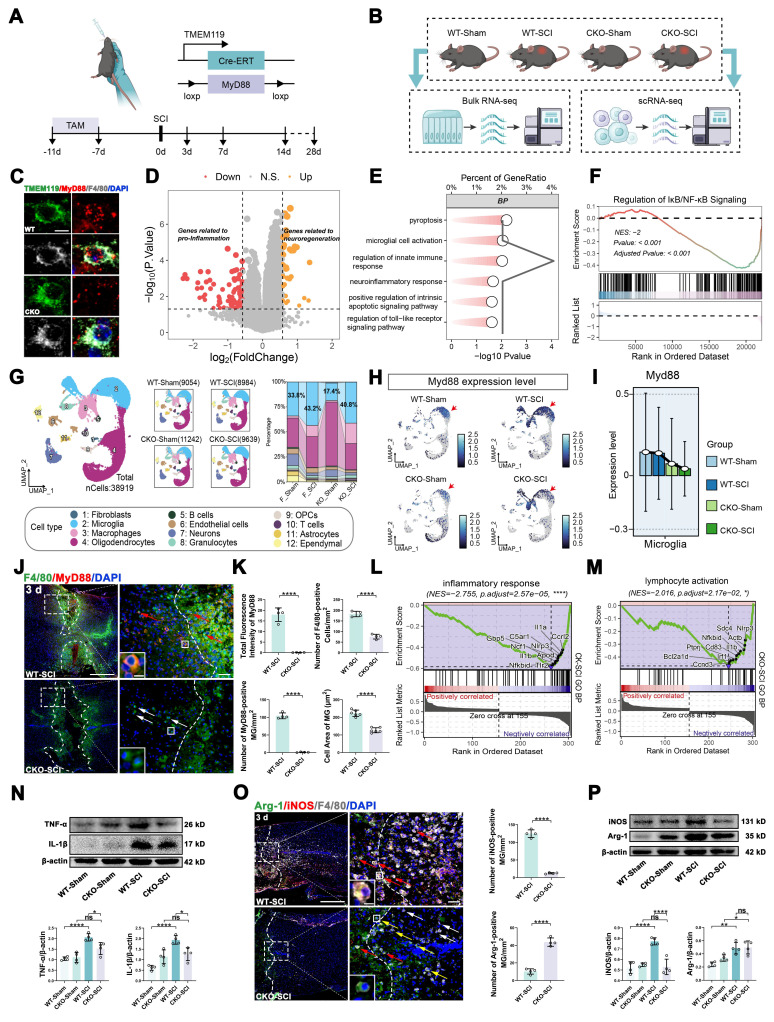
Microglia-specific MyD88 deletion suppresses inflammatory programs and accelerates recovery of homeostasis after SCI. (A) Breeding strategy for *Tmem119*^CreERT2^;*Myd88*^fl/fl^ mice and tamoxifen induction timeline. (B) Experimental design for bulk RNA-seq and scRNA-seq at 3 dpi in WT and cKO mice under sham or SCI conditions. (C) Representative immunofluorescence at 3 dpi showing TMEM119 (green), MyD88 (red), F4/80 (white), and DAPI (blue). Scale bar: 5 μm. (D) Volcano plot of DEGs (cKO-SCI vs. WT-SCI) from bulk RNA-seq. (E) Functional enrichment of DEGs highlighting reduced immune activation and TLR signaling in cKO-SCI. (F) GSEA showing depletion of “Regulation of IκB/NF-κB signaling” in cKO-SCI. (G) UMAP of 38,919 cells from scRNA-seq showing 12 major cell types and group-wise proportions. (H) Feature plots of *Myd88* expression across groups. (I) Quantification of *Myd88* expression in microglia across groups. (J) Representative sections at 3 dpi stained for F4/80 (green), MyD88 (red), and DAPI (blue). Dashed lines indicate lesion boundaries; boxed regions show higher magnification. (K) Quantification of MyD88 fluorescence intensity and F4/80^+^ cell parameters within the lesion region. Data are mean ± SD; n = 4 mice/group; unpaired two-tailed *t*-test. (L-M) GSEA showing reduced enrichment of inflammatory response and lymphocyte activation gene sets in cKO-SCI microglia. (N) Representative immunoblots and quantification of TNF-α and IL-1β at 3 dpi; normalized to β-actin. Data are mean ± SD; n = 4 mice/group; one-way ANOVA with Tukey’s post hoc test. (O) Representative sections at 3 dpi stained for Arg-1 (green), iNOS (red), F4/80 (white), and DAPI (blue). Data are mean ± SD; n = 4 mice/group; unpaired two-tailed *t*-test. (P) Representative immunoblots and quantification of iNOS and Arg-1; normalized to β-actin. Data are mean ± SD; n = 4 mice/group; one-way ANOVA with Tukey’s post hoc test. Scale bars: 500 μm (overview), 50 μm (insets), 10 μm (single-cell views) unless otherwise indicated. Statistical significance: *p < 0.05, **p < 0.01, ****p < 0.0001; ns, not significant.

To define the microglia-intrinsic contribution of MyD88 to SCI pathology, we generated inducible microglia-specific MyD88 conditional knockout mice by crossing *Tmem119*^CreERT2^ mice with *Myd88*^fl/fl^ mice (Figure 3A). After tamoxifen induction, MyD88 loss was restricted to TMEM119^+^ microglia, with *Myd88*^fl/fl^ littermates serving as WT controls (Figure 3C, Figure S2A-C). We analyzed spinal cord transcriptomes at 3 dpi under sham and SCI conditions (Figure 3B). Bulk RNA-seq revealed a clear separation between WT-SCI and cKO-SCI samples (Figure S3A), and identified 36 upregulated and 102 downregulated differentially expressed genes (DEGs) in cKO-SCI relative to WT-SCI spinal cords (Figure 3D). Functional enrichment analysis showed that genes upregulated in cKO-SCI were linked to axon regeneration and neural repair, whereas downregulated genes mapped to microglia activation, neuroinflammatory responses, and TLR signaling (Figure 3E; Figure S3B). GSEA further showed suppression of NF-κB signaling and relative enrichment of neurogenesis in cKO-SCI tissue (Figure 3F; Figure S3C).

We next used scRNA-seq to characterize cellular composition and microglial programs at single cell resolution across four groups (WT-Sham, WT-SCI, cKO-Sham, cKO-SCI), which produced 38,919 cells and 12 major cell types after quality control and clustering (Figure 3G; Figure S3D) 12. Relative to WT-SCI, the microglial proportion dropped by ~2.4% in cKO-SCI (Figure 3G). Quantitative analysis showed that the average *Myd88* expression level in cKO-SCI microglia was reduced by 70.98% compared with WT-SCI (0.0398 vs. 0.1370 normalized UMI), while expression in all other cell types remained unchanged between groups (Figure 3H-I; Figure S3E). UMAP feature plots and violin plots of apoptosis score in microglia revealed no significant difference between cKO-SCI and WT-SCI groups (Figure S3F), ruling out the possibility that the observed state shift was driven by selective cell death.

We next validated these molecular changes at the tissue level at 3 dpi. Immunofluorescence showed reduced MyD88 signal within and around the lesion epicenter in cKO-SCI mice compared with WT-SCI controls (Figure 3J), accompanied by fewer MyD88^+^F4/80^+^ cells and reduced F4/80^+^ cell density in the lesion region (Figure 3K). In microglia, pathway-level analyses further supported attenuation of inflammatory response and immune activation programs in cKO-SCI, including reduced enrichment of leukocyte activation-related gene sets (Figure 3L-M; Figure S3G). Consistent with transcriptomic findings, immunoblotting at 3 dpi showed lower TNF-α and IL-1β protein levels in cKO-SCI spinal cords (Figure 3N).

Finally, we examined microglia activation markers at the lesion site. Compared with WT-SCI, cKO-SCI spinal cords exhibited reduced iNOS immunoreactivity and increased Arg-1 fluorescence intensity, with a higher number of Arg-1^+^ microglia in the lesion region (Figure 3O). Immunoblotting corroborated reduced iNOS protein levels in cKO-SCI, while Arg-1 protein levels were comparable between groups (Figure 3P). In addition, TMEM119^+^ homeostatic microglia reappeared earlier in the lesion area of cKO mice than in that of WT mice (Figure S4A-B). Together, these data demonstrate that microglial MyD88 deletion suppresses acute inflammatory signaling and accelerates recovery of homeostatic microglial features after SCI.

### Microglia-specific MyD88 deletion shifts microglial states toward a migrating program and is associated with improved repair and functional recovery

**Figure 4:**
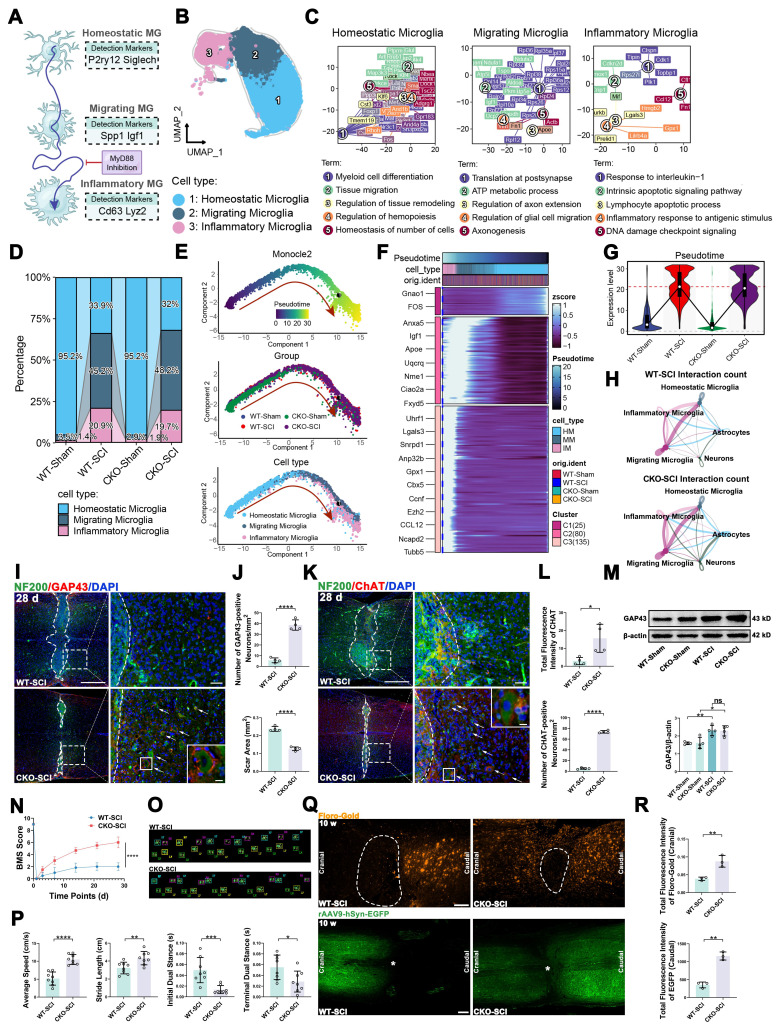
Microglial MyD88 deletion shifts injury-activated microglial states and improves tissue repair, axonal growth, and locomotor recovery. (A) Schematic of microglial state transitions after SCI and the proposed impact of MyD88 inhibition. (B) UMAP of microglia showing three states: homeostatic (*P2ry12*, *Siglech*), migrating (*Spp1*, *Igf1*), and inflammatory (*Cd63*, *Lyz2*). (C) Representative functional enrichment terms for each microglial state. (D) State proportions across groups during the acute phase. (E) Monocle2 pseudotime trajectories. (F) Heatmap of genes dynamically regulated along pseudotime. (G) Distribution of cells along pseudotime across groups. (H) Ligand-receptor interaction analysis of intercellular communication among homeostatic, inflammatory, migrating microglia, neurons and astrocytes in WT-SCI and cKO-SCI mice. (I) Representative sections at 28 dpi stained for NF200 (green), GAP43 (red), and DAPI (blue). (J) Quantification of GAP43^+^ neurons and scar area at 28 dpi. Data are mean ± SD; n = 4 mice/group; unpaired two-tailed *t*-test. (K) Representative ventral horn sections at 28 dpi stained for ChAT (red), NF200 (green), and DAPI (blue). (L) Quantification of ChAT^+^NF200^+^ neurons at 28 dpi. Data are mean ± SD; n = 4 mice/group; unpaired two-tailed *t*-test. (M) Representative immunoblots and quantification of GAP43 at 28 dpi; normalized to β-actin. Data are mean ± SD; n = 4 mice/group; one-way ANOVA with Tukey’s post hoc test. (N) BMS scores after SCI. Data are mean ± SD; n = 6 mice/group; two-way repeated-measures ANOVA. (O) Representative CatWalk footprints at 28 dpi. (P) Quantification of CatWalk gait parameters at 28 dpi. Data are mean ± SD; n = 8 mice/group; unpaired two-tailed *t*-test. (Q) Representative images of retrograde Fluoro-Gold and anterograde rAAV9-hSyn-EGFP tracing. Dashed lines indicate lesion borders; asterisks indicate scar regions. Scale bars: 100 μm. (R) Quantification of Fluoro-Gold signal cranial to the lesion and EGFP signal caudal to the lesion. Data are mean ± SD; n = 3 mice/group; unpaired two-tailed *t*-test. Scale bars: 500 μm (overview), 50 μm (insets), 10 μm (single-cell views) unless otherwise indicated. Statistical significance: *p < 0.05, **p < 0.01, ***p < 0.001, ****p < 0.0001; ns, not significant.

Given the dynamic state transitions of microglia after SCI, we investigated whether microglial MyD88 deletion reshapes the microglial state composition and is related to repair outcomes (Figure 4A). Based on published cell-state annotations 12, microglia were classified into three transcriptional states: homeostatic (*P2ry12*, *Siglech*), migrating (*Spp1*, *Igf1*), and inflammatory (*Cd63*, *Lyz2*) (Figure 4B; Figure S5A). Functional enrichment analysis indicated that homeostatic microglia were enriched for tissue homeostasis and remodeling terms, migrating microglia for cell migration and axonogenesis, and inflammatory microglia for inflammatory activation and apoptosis-associated responses (Figure 4C).

In the acute phase, cKO-SCI spinal cords exhibited a modestly increased proportion of migrating microglia and a reduced proportion of inflammatory microglia compared with WT-SCI (Figure 4D). The cell numbers and proportions for each subpopulation across groups are summarized in Table S2. We used pseudotime trajectory analysis to model state transitions. Microglia were arranged along a continuum from homeostatic to injury-associated states, progressing through a migrating intermediate toward an inflammatory endpoint (Figure 4E-F). Of note, cKO-SCI microglia were redistributed along this trajectory, with reduced representation at the inflammatory end and increased representation toward the migrating segment (Figure 4G). We next asked whether the observed shift in microglial states was functionally significant by performing ligand-receptor interaction analysis. This analysis showed stronger intercellular communication between the migrating and inflammatory subpopulations, as well as between migrating microglia and neurons, in cKO-SCI compared with WT-SCI (Figure 4H), indicating that the proportional redistribution is sufficient to produce biologically meaningful changes in microglial crosstalk.

We next assessed whether this state shift was accompanied by improved tissue repair. Immunofluorescence analyses at 14 and 28 dpi showed a higher TMEM119^+^ signal in peri-lesional regions in cKO-SCI than in WT-SCI at both time points (Figure S5B-C), consistent with sustained recovery of homeostatic features. At 28 dpi, peri-lesional gray matter in cKO-SCI mice displayed a stronger NF200 signal, increased GAP43 immunoreactivity near the lesion border, and better-preserved neuronal morphology (Figure 4I-J). In the ventral horn, cKO-SCI mice exhibited a higher number of ChAT^+^NF200^+^ motor neurons (Figure 4K-L). TUJ1/synaptophysin staining further showed increased neuronal and synaptic puncta density around the lesion (Figure S5C). In parallel, the scar area at 28 dpi was reduced in cKO-SCI relative to WT-SCI (Figure 4J; Figure S5D), and immunoblotting confirmed increased GAP43 protein abundance (Figure 4M). Fibronectin (encoded by *Fn1*), an extracellular matrix protein implicated in tissue repair and axonal guidance, revealed sustained perilesional expression in cKO-SCI microglia compared with WT-SCI (Figure S6A-B). Functionally, cKO-SCI mice exhibited improved hindlimb locomotor recovery by BMS scoring (Figure 4N) and superior gait parameters by CatWalk analysis at 28 dpi (Figure 4O-P). Finally, bidirectional tracing at 10 weeks post-injury showed increased Fluoro-Gold labeling cranial to the lesion and enhanced rAAV9-hSyn-EGFP signal caudal to the lesion in cKO-SCI mice (Figure 4Q-R; Figure S5E), indicating enhanced long-range axonal connectivity. Consistent with this, CMAP recordings at the same time point revealed significantly improved amplitude and reduced latency in cKO-SCI mice compared with WT-SCI controls (Figure S7), providing functional evidence for improved axonal conductivity across the lesion site.

### TGF-β signaling is required for the protective phenotype associated with microglial MyD88 deletion

**Figure 5:**
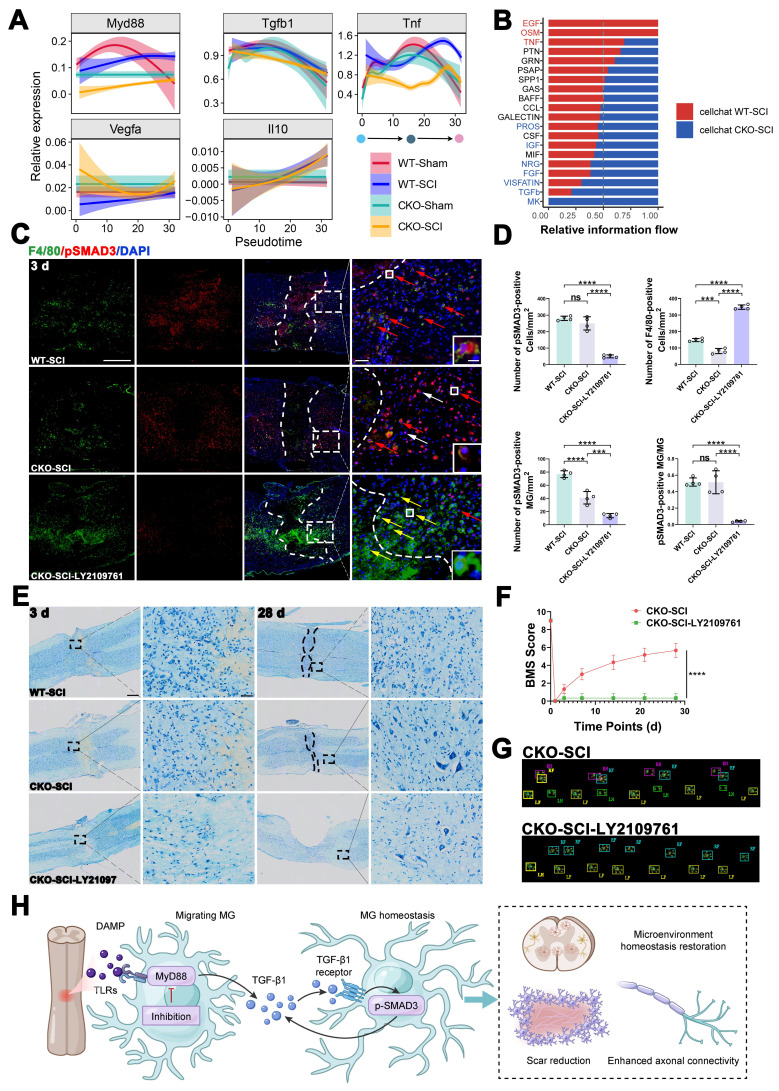
TGF-β1 receptor/pSMAD3 signaling is required for microglial state transition and functional recovery in microglial MyD88 cKO mice. (A) Smoothed expression of *Myd88*, *Tgfb1*, *Tnf*, *Vegfa*, and *Il10* along microglial pseudotime across groups. (B) CellChat analysis of differential intercellular communication networks between WT-SCI and cKO-SCI groups. Upregulated signaling pathways in cKO-SCI (e.g., FGF, TGF-β) and WT-SCI (e.g., EGF, OSM, TNF) are shown. Bar length represents the relative signaling strength. (C) Representative sections at 3 dpi stained for F4/80 (green), pSMAD3 (red), and DAPI (blue). Dashed lines indicate lesion regions; boxed regions show higher magnification. (D) Quantification of pSMAD3^+^ cells and microglia responses at 3 dpi. Data are mean ± SD; n = 4 mice/group; one-way ANOVA with Tukey’s post hoc test. (E) Representative Nissl staining at 3 and 28 dpi. Scale bars: 400 μm (overview), 40 μm (enlarged). (F) BMS scores comparing cKO-SCI and cKO-SCI + LY2109761 groups. Data are mean ± SD; n = 6 mice/group; two-way repeated-measures ANOVA. (G) Representative CatWalk footprints at 28 dpi. (H) Working model linking microglial MyD88 suppression to TGF-β-dependent repair. Scale bars: 500 μm (overview), 50 μm (insets), 10 μm (single-cell views) unless otherwise indicated. Statistical significance: ***p < 0.001, ****p < 0.0001; ns, not significant.

In search of candidate mediators downstream of microglial MyD88 deletion, we examined gene dynamics along the microglial pseudotime trajectories. *Myd88* expression remained reduced in cKO-SCI microglia throughout pseudotime, including late pseudotime corresponding to inflammatory states (Figure 5A). In parallel, *Tnf* exhibited a late pseudotime peak in WT-SCI microglia that was blunted in cKO-SCI microglia (Figure 5A). By comparison, *Tgfb1* displayed higher expression in cKO-SCI microglia across pseudotime (Figure 5A), pointing to enhanced TGF-β signaling as a candidate pathway associated with the state shift. CellChat analysis showed stronger reparative signaling (FGF, TGF-β) and weaker pro-inflammatory signaling (EGF, OSM, TNF) in cKO-SCI compared with WT-SCI (Figure 5B), and the prominent TGF-β upregulation aligned with increased *Tgfb1* expression along the microglial pseudotime trajectory. We next analyzed the expression profiles of *Tgfb1* and its receptors (*Tgfbr1*, *Tgfbr2*, *Acvr1*) across major cell types to clarify the cellular source and targets of TGF-β signaling. *Tgfb1*, *Tgfbr1* and *Tgfbr2* were predominantly expressed by microglia, whereas *Acvr1* was expressed in both neurons and microglia. Astrocytes showed negligible expression of TGF-β signaling components in our dataset (Figure S8A). Within the microglial compartment, homeostatic and migrating microglia exhibited higher expression of *Tgfb1*, *Tgfbr1* and *Tgfbr2* than inflammatory microglia, implying that at 3 dpi, microglia serve as both the principal source and the primary target of TGF-β1.

We then tested the functional relevance of TGF-β signaling by administering the TGF-β receptor inhibitor LY2109761 to cKO mice immediately after SCI for 3 consecutive days (Figure S8B). We used nuclear pSMAD3 as a downstream readout of TGF-β signaling at 3 dpi (Figure 5C) 33. Compared with WT-SCI, the absolute number of pSMAD3^+^F4/80^+^ cells was reduced in cKO-SCI spinal cords; however, the fraction of pSMAD3^+^ cells within the F4/80^+^ population did not differ between groups (Figure 5D). Meanwhile, cKO-SCI tissue exhibited an increased nuclear pSMAD3 signal in peri-lesional non-microglia (Figure 5C). Importantly, LY2109761 markedly reduced pSMAD3 signal (Figure 5C-D), and increased the density of F4/80^+^ cells with a more activated morphology (Figure 5C-D), indicating the failure of the anti-activation mechanism of microglia in cKO mice without TGF-β signaling intervention. Ligand-receptor analysis further confirmed *Tgfb1*-*Tgfbr1*/*Tgfbr2* as the dominant signaling axis, with *Acvr1* contributing weakly (Figure S8C). Together with the predominant microglial expression of *Tgfbr1*/*Tgfbr2* (Figure S8A) and the active inter-subpopulation crosstalk observed after SCI (Figure 4H), these data establish a TGF-β autocrine loop as the primary mechanism by which MyD88-deficient microglia regulate their reprogrammed state.

To ask whether TGF-β signaling contributes to tissue preservation and functional recovery in cKO mice, we performed Nissl staining and behavioral tests. Peri-lesional neuronal morphology was better preserved in cKO-SCI mice than WT-SCI at 3 and 28 dpi, whereas LY2109761-treated cKO-SCI mice showed pronounced neuronal loss and tissue disruption (Figure 5E). In agreement with these histological changes, LY2109761 abolished locomotor improvement in cKO-SCI mice as measured by longitudinal BMS scoring and CatWalk analysis (Figure 5F-G). These results indicate that intact TGF-β signaling is required for the tissue-protective and functional benefits associated with microglial MyD88 deletion (Figure 5H).

### Microglia-targeted biomimetic nanoparticles enable lesion-retained delivery of ST2825 and improve recovery after SCI

**Figure 6:**
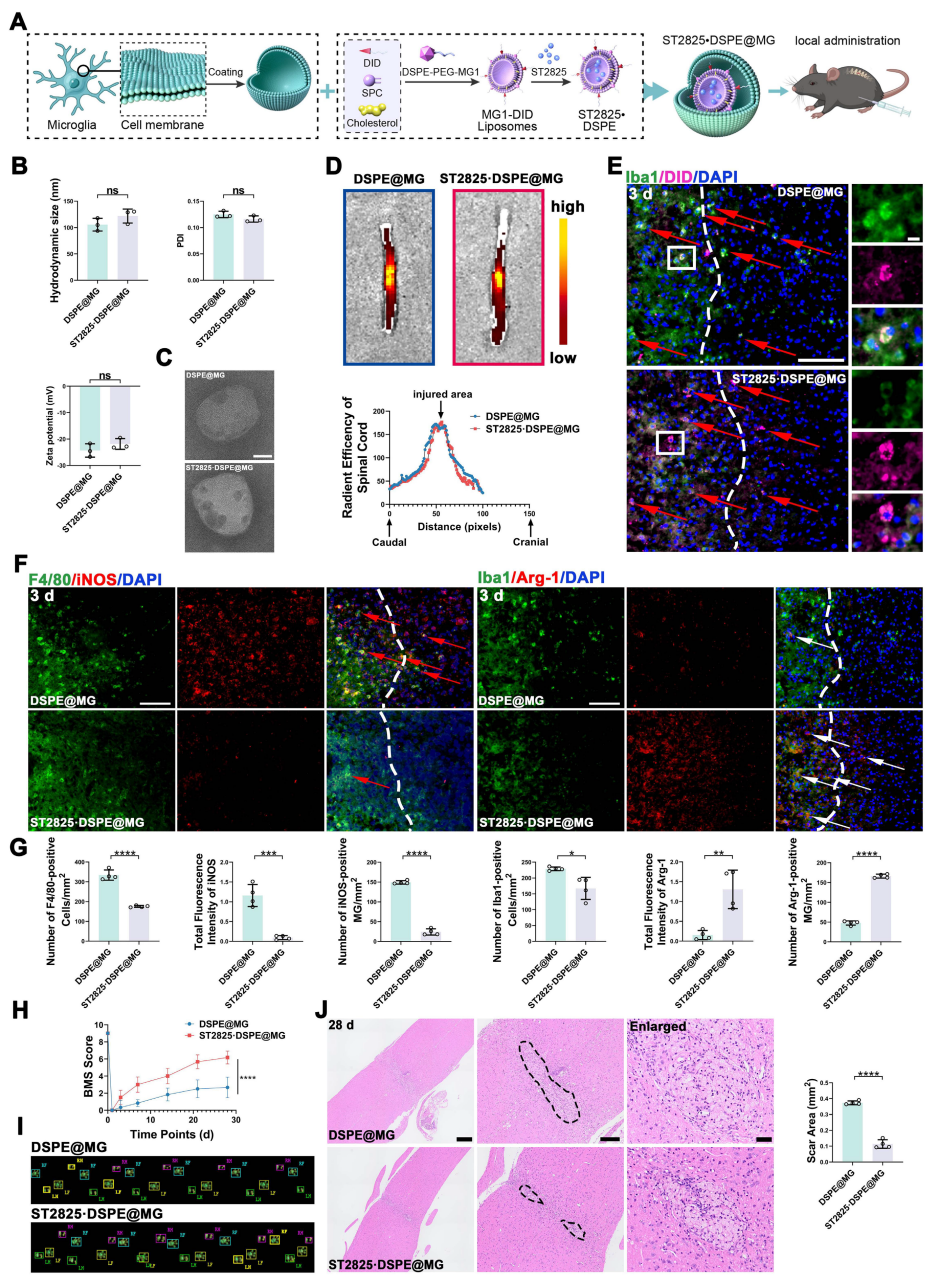
Microglia-targeted ST2825·DSPE@MG nanoparticles show microglia uptake and therapeutic efficacy after SCI. (A) Schematic of ST2825·DSPE@MG fabrication. (B) DLS characterization of DSPE@MG and ST2825·DSPE@MG (hydrodynamic diameter, PDI, zeta potential). Data are mean ± SD; n = 3; unpaired two-tailed *t*-test. (C) Representative TEM images showing core-shell morphology. Scale bars: 50 nm. (D) *Ex vivo* fluorescence imaging at 3 days after administration of DiD-labeled nanoparticles and radiant efficiency profiles along the rostrocaudal axis. (E) Images showing colocalization of DiD-labeled nanoparticles with Iba1^+^ microglia at the lesion boundary. Scale bars: 100 μm (overview) and 10 μm (single-cell views). (F) Representative sections at 3 dpi stained for F4/80 (green) and iNOS (red) (left), and Iba1 (green) and Arg-1 (red) (right); DAPI (blue). Scale bars: 100 μm. (G) Quantification of lesion-region microglia and markers at 3 dpi. Data are mean ± SD; n = 4 mice/group; unpaired two-tailed *t*-test. (H) BMS scores after SCI. Data are mean ± SD; n = 6 mice/group; two-way repeated-measures ANOVA. (I) Representative CatWalk footprints at 28 dpi. (J) Representative H-E staining at 28 dpi and quantification of scar area. Data are mean ± SD; n = 4 mice/group; unpaired two-tailed *t*-test. Scale bars: 400 μm (overview), 100 μm (injury area), and 40 μm (enlarged). Statistical significance: *p < 0.05, **p < 0.01, ***p < 0.001, ****p < 0.0001; ns, not significant.

To enhance the delivery of ST2825 to the injured spinal cord and promote microglia uptake, we developed microglia membrane-coated, MG1 peptide-modified biomimetic nanoparticles (ST2825·DSPE@MG) (Figure 6A) 34. Dynamic light scattering (DLS) showed that DSPE@MG and ST2825·DSPE@MG had comparable hydrodynamic diameters (~90-140 nm) with low polydispersity (PDI < 0.2) and mildly negative zeta potentials (~-15 to -30 mV), indicating stable and monodisperse formulations (Figure 6B). Transmission electron microscopy (TEM) revealed spherical particles with a core-shell morphology consistent with membrane coating (Figure 6C). *In vitro* drug release assays demonstrated that ST2825·DSPE@MG exhibited sustained release of ST2825 over 72 h and achieved ~70% cumulative release (Figure S9A). Colloidal stability tests confirmed that particle size, PDI and zeta potentials remained stable at 4 ℃ over 14 days (Figure S9A). Size distribution by intensity confirmed a unimodal distribution for both DSPE@MG and ST2825·DSPE@MG, and no appreciable peak shift or broadening after drug loading (Figure S9B).

We next assessed lesion targeting using DiD-labeled nanoparticles following local administration. *Ex vivo* fluorescence imaging at 3 dpi showed strong DiD signals concentrated at the injury epicenter in both DSPE@MG and ST2825·DSPE@MG groups (Figure 6D). Radiant efficiency profiles along the rostrocaudal axis peaked at the lesion segment and declined into peri-lesional regions (Figure 6D). *In vivo* fluorescence imaging from 1 to 7 days post-administration further confirmed that the DiD-labeled nanoparticle signal remained strictly confined to the lesion segment without detectable leakage to adjacent segments or systemic distribution (Figure S9C). Imaging revealed extensive colocalization of DiD-labeled nanoparticles with Iba1^+^ microglia at the lesion boundary, with a punctate intracellular DiD signal (Figure 6E). Quantitative analysis showed that ~65% of Iba1⁺ microglia in the lesion area were DiD⁺, with nearly all detectable DiD signal colocalizing with Iba1 (Figure S9D), demonstrating efficient and selective microglial uptake of the nanoparticles. We also prepared and characterized plain liposomes loaded with ST2825 as an additional control. They exhibited a uniform size distribution, good colloidal stability, high drug loading capacity (6.25%) and encapsulation efficiency (93.75%), and spherical morphology under TEM (Figure S9E-F). However, these plain liposomes did not produce a noticeable behavioral improvement (Figure S9G).

We then investigated whether microglia-targeted delivery of ST2825 modulates early neuroinflammatory responses after SCI. At 3 dpi, ST2825·DSPE@MG treatment reduced the number of F4/80^+^ cells and decreased iNOS signal within the lesion region compared with DSPE@MG controls (Figure 6F-G). At the same time, Arg-1 signal and the number of Arg-1^+^Iba1^+^ cells were increased in the ST2825·DSPE@MG group (Figure 6F-G), reflecting a shift in marker expression patterns within the lesion microenvironment.

Lastly, we evaluated tissue remodeling and functional recovery. BMS scoring showed improved locomotor performance in ST2825·DSPE@MG-treated mice compared with DSPE@MG controls (Figure 6H), and CatWalk analysis at 28 dpi further confirmed an improved gait performance (Figure 6I). H-E staining at 28 dpi demonstrated a reduced scar area in the ST2825·DSPE@MG group (Figure 6J). Together, these data indicate that biomimetic nanoparticle delivery of ST2825 enhances microglia uptake and improves functional recovery after SCI. Similar to the findings in females, ST2825·DSPE@MG treatment in males promoted the transition of lesion-associated microglia from a pro-inflammatory toward a repair-associated phenotype (Figure S10A-B).

## Discussion

SCI elicits a rapid and sustained innate immune response that drives secondary tissue damage and constrains functional recovery 35. MyD88 integrates signals from TLRs and acts as a proximal adaptor for NF-κB-dependent inflammatory transcription. Previous studies have demonstrated that global inhibition of MyD88 signaling can attenuate inflammation following CNS injury 36, 37. However, such broad-spectrum interventions have key limitations: they mask the cell-type-specific functions of MyD88 and fail to actively drive injury-activated microglia toward a reparative state. The incremental contributions of the present study are threefold: (1) microglia-specific MyD88 conditional knockout was achieved for the first time in the context of SCI, revealing that microglia-intrinsic MyD88 signaling governs pathological state transitions; (2) TGF-β signaling was identified as a functional downstream bridge linking MyD88 suppression to tissue repair; and (3) a biomimetic nanodelivery strategy was developed based on this mechanistic understanding. Collectively, this work represents a paradigm shift from broad-spectrum anti-inflammatory intervention to precision microglial reprogramming.

A key finding of this study is that MyD88 signaling is activated shortly after SCI and remains elevated throughout the acute-to-subacute phase. Bulk transcriptomics and immunoblotting indicated sustained activation of the TLRs-MyD88 axis from hours to days after injury. Interrogation of the Tabulae Paralytica single-cell atlas further localized high *Myd88* expression to microglia, indicating that MyD88 induction after SCI was not uniformly distributed across spinal cord cell types. This temporal and cellular resolution has translational relevance, as the clinical failure of anti-inflammatory strategies in SCI has been attributed, in part, to imprecise treatment windows and inadequate cellular specificity, which can suppress beneficial immune functions while failing to target dominant pathological drivers 38-41. Consistent with prolonged innate immune activation in chronic SCI 42, AUCell scoring suggested persistent TLRs-MyD88 pathway activity in microglia over weeks. Endothelial enrichment of *Myd88* suggested potential vascular contributions. Nevertheless, our microglia-specific genetic studies indicated that microglial MyD88 was a dominant determinant of pathological outcomes, aligning with emerging evidence that microglia actively regulate lesion microenvironments and scar dynamics rather than serving as passive responders 43-45.

The second major finding is that MyD88 activity is coupled with microglial state remodeling after SCI. Across datasets, *Myd88* expression showed a weak association with homeostatic markers but aligned with inflammatory and repair-associated marker sets, suggesting that MyD88 engagement accompanied state transitions rather than resting homeostasis. *In vitro*, two independent inhibitory approaches (MIP in N9 cells and ST2825 in primary microglia) converged on a consistent phenotype: reduced LPS-induced iNOS expression and decreased pro-inflammatory cytokine release (TNF-α, IL-1β), with preservation of trophic mediators (IL-10, CSF-1, IGF-1, TGF-β1). These findings support the concept that MyD88 inhibition does not simply silence microglial activity but actively reprograms it toward a less damaging, potentially pro-repair state. *In vivo*, local ST2825 administration confirmed these features during the acute phase, including reduced iNOS^+^ microglia, increased Arg-1 labeling, and improved perilesional neuronal preservation. Importantly, early immunomodulation can have opposing consequences depending on timing and cellular specificity: some components of the glial response, including aspects of scar formation, can stabilize injured tissue and limit lesion spread 46, 47. Our results therefore support a microglia-targeted framework in which dampening maladaptive signaling while preserving protective functions may be more effective than indiscriminate immunosuppression.

Using *Tmem119*^CreERT2^;*Myd88*^fl/fl^ mice, we tested microglia-intrinsic MyD88 function while reducing confounding from peripherally derived myeloid cells. Although TMEM119 expression can be downregulated after SCI, this concern was mitigated by completing tamoxifen induction prior to injury with a 7-day washout period. This design ensured that CreERT2-mediated recombination primarily targeted homeostatic microglia before injury onset. Our scRNA-seq data confirmed that *Myd88* downregulation was strictly confined to microglia across all 12 major cell types analyzed. At 3 dpi, bulk RNA-seq demonstrated that microglial MyD88 deletion shifted the injured spinal cord transcriptome away from innate immune activation and toward axon growth and repair-associated programs. scRNA-seq further confirmed efficient *Myd88* reduction in microglia and revealed attenuation of inflammatory response and immune activation gene sets, including reduced enrichment of lymphocyte activation-related programs. These results suggest that microglial MyD88 contributes not only to intrinsic inflammatory amplification but also to broader immune network activation within the lesion environment. Tissue-level validation corroborated these transcriptomic findings, including reduced MyD88/F4/80 co-labeling and lower TNF-α and IL-1β protein levels. Together, these data position microglial MyD88 upstream of early inflammatory amplification that likely contributes to secondary injury cascades. They further support a model in which MyD88 signaling delays resolution by sustaining injury-associated inflammatory programs, consistent with its established role in TLR signaling and NF-κB activation. Importantly, our state-based and trajectory-based analyses provide a framework that aligns with contemporary single-cell literature, which emphasizes microglial continua rather than binary “M1/M2” endpoints.

Beyond molecular signatures, our study links microglial MyD88 deletion to structural and functional outcomes. By classifying microglia into homeostatic, migrating and inflammatory states, we observed that cKO mice showed a reduced fraction of inflammatory microglia and an increased fraction of migrating microglia during the acute phase. Pseudotime analysis supported a continuum from homeostatic states through a migrating intermediate toward inflammatory endpoints, and MyD88 deletion redistributed cells away from inflammatory termini. This state redistribution was accompanied by a reduced scar area, enhanced GAP43 expression, improved preservation of neuronal markers, and increased synaptic puncta around the lesion. Functionally, these anatomical correlations translated into improved BMS scores and CatWalk gait parameters. Notably, bidirectional tracing and CMAP electrophysiology provided convergent anatomical and functional evidence for enhanced axonal connectivity across the lesion, a stringent and clinically relevant endpoint 48-50. While astrocytes and fibroblasts are canonical drivers of scar formation, microglia modulate scar architecture and extracellular matrix dynamics through cytokine signaling, phagocytic clearance, and crosstalk with astrocytes and infiltrating immune cells 51-53. Our data identify MyD88 as a microglial node linking innate immune signaling to tissue-scale remodeling after SCI.

A central mechanistic insight is that the protective phenotype associated with microglial MyD88 deletion requires intact TGF-β signaling. Along the microglial pseudotime trajectory, cKO microglia exhibited elevated *Tgfb1* expression and attenuated *Tnf* induction relative to WT, indicating a shift toward a TGF-β-permissive signaling environment. CellChat analysis confirmed a shift from pro-inflammatory to reparative intercellular signaling networks in cKO-SCI mice, further supporting the central role of TGF-β signaling in the protective phenotype. Pharmacological TGF-β receptor blockade with LY2109761 suppressed pSMAD3 expression and reversed tissue preservation and locomotor benefits in cKO mice, establishing pathway necessity. Notably, rather than passively dampening inflammation, MyD88-deficient microglia appear to actively rewire their signaling from pro-inflammatory outputs to autocrine TGF-β homeostatic reinforcement. This self-sustaining mechanism may explain the durability of the reprogrammed state, as the autocrine feedback loop could maintain microglial homeostasis beyond the initial injury phase. The enhanced crosstalk between migrating microglia and neurons further suggests that TGF-β signaling facilitates neuron-microglia communication, contributing to a pro-regenerative microenvironment. Although the absolute number of pSMAD3⁺ microglia decreased in cKO tissue, nuclear pSMAD3 signal was increased in perilesional non-microglia, suggesting that microglial MyD88 deletion reshapes the inflammatory microenvironment and redistributes SMAD3 activation across cell types. These findings align with previous reports implicating TGF-β signaling in limiting inflammation, promoting resolution, and supporting CNS repair, including the maintenance of microglial identity 33, 54-57.

To translate mechanistic insights into a therapeutically actionable modality, we engineered a microglia-targeted biomimetic nanoparticle platform (ST2825·DSPE@MG) that enhanced lesion-localized delivery and microglia uptake of ST2825. This delivery strategy strengthened modulation of microglia activation markers, improved tissue preservation, and enhanced locomotor recovery compared with control nanoparticles. Biomimetic membrane coating and peptide-mediated targeting have emerged as complementary strategies to improve cellular specificity and retention in complex injury microenvironments 34. By integrating these principles for microglia-oriented delivery, our platform shows that microglia-targeted MyD88 modulation can be packaged into a translationally relevant strategy for SCI.

Several limitations should be acknowledged. First, the crush injury model does not permit absolute discrimination between spared and regenerated axons; future studies employing complete transection models and fluorescence-based labeling strategies will be required for rigorous distinction. Second, the majority of experiments were conducted on female mice. Although ancillary experiments confirmed that the core pharmacodynamic effects of ST2825·DSPE@MG are similar in males, dedicated studies with larger male cohorts are warranted. Third, it should be noted that the present study adopted DSPE@MG as the core control to address the primary proof-of-concept question of whether therapeutic efficacy is attributable to MyD88 inhibition. Comprehensive *in vivo* tissue distribution analysis, complete PK/PD evaluation, and immunogenicity assessment will be essential components of subsequent preclinical development. Fourth, rigorous validation of the nanoparticle targeting advantage will require stepwise dissection of the respective contributions of the membrane coating and the MG1 peptide. Fifth, while our data support a model in which autocrine TGF-β signaling plays the dominant role in the protective phenotype, cell-type-specific TGF-β receptor knockout tools will be needed to precisely dissect autocrine versus paracrine contributions. Sixth, the scRNA-seq dataset lacked biological replicates for each condition; therefore, differences in cell-type proportions should be interpreted cautiously. Finally, we note that F4/80 labels both activated resident microglia and infiltrating macrophages in the injured spinal cord; accordingly, F4/80⁺ cells are more accurately interpreted as "F4/80⁺ myeloid cells" throughout this study; and the "migrating microglia" designation reflects transcriptomic classification rather than a direct inference of *in vivo* motility.

## Conclusions

This study identifies microglial MyD88 as an early and persistent regulator of injury-activated microglia remodeling following SCI. Microglia-specific MyD88 deletion rebalances microglial state trajectories toward repair-associated programs, accompanied by reduced neuroinflammation, accelerated recovery of homeostasis, reduced scarring, enhanced axonal connectivity, and improved locomotor function. Mechanistically, these benefits require intact TGF-β signaling. Finally, microglia-targeted biomimetic nanoparticles enable localized MyD88 inhibition with therapeutic benefits, providing a translational route to leverage state-based immunomodulation in SCI.

## Supplementary Material

Supplementary figures and tables.

## Data Availability

The datasets supporting the conclusions of this article are included within the article and its additional files (Figs. S1-S10, Table S1-S2).

## Abbreviations

SCI: Spinal cord injury
MyD88: Myeloid differentiation primary response 88
TLR: Toll-like receptor
NF-κB: Nuclear factor kappa-light-chain-enhancer of activated B cells
TGF-β: Transforming growth factor beta
cKO: Conditional knockout
WT: Wild-type
BMS: Basso Mouse Scale
ROIs: regions of interest
PK/PD: pharmacokinetic/pharmacodynamic
GAP43: Growth-associated protein 43
Iba1: Ionized calcium-binding adapter molecule 1
iNOS: Inducible nitric oxide synthase
Arg-1: Arginase-1
DSPE: 1,2-Distearoyl-sn-glycero-3-phosphoethanolamine
CMAP: compound muscle action potential
DEG: Differentially expressed gene
DLS: Dynamic light scattering
GSEA: Gene set enrichment analysis
scRNA-seq: Single-cell RNA sequencing
UMAP: Uniform manifold approximation and projection
