# Supplementary material for: Targeting microglial MyD88 reprograms injury-activated microglia and promotes spinal cord repair via TGF-β signaling and biomimetic nanodelivery
Source: Theranostics. 2026 Jul 13;16(14):8017–35. doi: 10.7150/thno.136733 (PMC13440466; doi:10.7150/thno.136733)
Supplement: Supplementary file 1 [file thnov16p8017s1.pdf]

Table S1.

Antibodies used in immunohistochemistry (IF) and Western blotting (WB)

Antigen	Host Species	Applicati-on	Vendor	Catalog No.	Dilution (IF)	Dilution (WB)
Primary Antibodies						
MyD88	Mouse	IF, WB	Proteintech	67969-1-Ig	1:400	1:2000
Iba1	Rabbit	IF	Wako	019-19741	1:800	—
F4/80	Rat	IF	Invitrogen	14-4801-82	1:100	—
GFAP	Mouse	IF	Millipore	MAB360	1:1000	—
iNOS (NOS2)	Rabbit	IF, WB	Proteintech	22226-1-AP	1:200	1:1000
Arg-1 (Arginase-1)	Mouse	IF, WB	Invitrogen	MA5-31577	1:200	1:1000
TMEM119	Rabbit	IF	Proteintech	27585-1-AP	1:400	—
pSMAD3	Rabbit	IF	Abcam	ab52903	1:200	—
NF200 (Neurofilament-H)	Mouse	IF	Proteintech	60331-1-Ig	1:400	—
GAP43	Rabbit	IF, WB	Proteintech	16971-1-AP	1:400	1:2000
ChAT	Rabbit	IF	Proteintech	20747-1-AP	1:200	—
TUJ1 (β-III tubulin)	Mouse	IF	Proteintech	66240-1-Ig	1:400	—
Synaptophysin (Syn)	Rabbit	IF	Proteintech	17785-1-AP	1:200	—
TLR4	Rabbit	WB	Abgent	AP1504a	—	1:500
TNF-α	Rabbit	WB	Abcam	ab6671	—	1:200
IL-1β	Mouse	WB	Cell Signaling	CS12242	—	1:1000
β-actin	Mouse	WB	Sigma-Aldrich	A5441	—	1:1000
Secondary Antibodies						
Donkey anti-Rabbit IgG, Alexa Fluor 488	Donkey	IF	Invitrogen	A21206	1:1000	—
Donkey anti-Rabbit IgG, Alexa Fluor 594	Donkey	IF	Invitrogen	A21207	1:1000	—
Goat anti-Mouse IgG, Alexa Fluor 488	Goat	IF	Invitrogen	A11001	1:1000	—
Goat anti-Mouse IgG, Alexa Fluor 594	Goat	IF	Invitrogen	A11032	1:1000	—
Goat Anti-Rat IgG, Alexa Fluor 647	Goat	IF	Abcam	ab150159	1:800	—
Goat anti-Rabbit IgG, HRP-conjugated	Goat	WB	Zhuangzhibio	EK020	—	1:1000
Goat anti-Mouse IgG, HRP-conjugated	Goat	WB	Zhuangzhibio	EK010	—	1:1000

Table S2

Corresponding cell numbers and proportions for each microglial subtype

Group	Total	Homeostatic	Migrating	Inflammatory
WT-Sham	3011	2867 (95.2%)	105 (3.5%)	42 (1.4%)
WT-SCI	3860	1309 (33.9%)	1745 (45.2%)	807 (20.9%)
cKO-Sham	1929	1836 (95.2%)	56 (2.9%)	37 (1.9%)
cKO-SCI	3882	1242 (32.0%)	1871 (48.2%)	765 (19.7%)

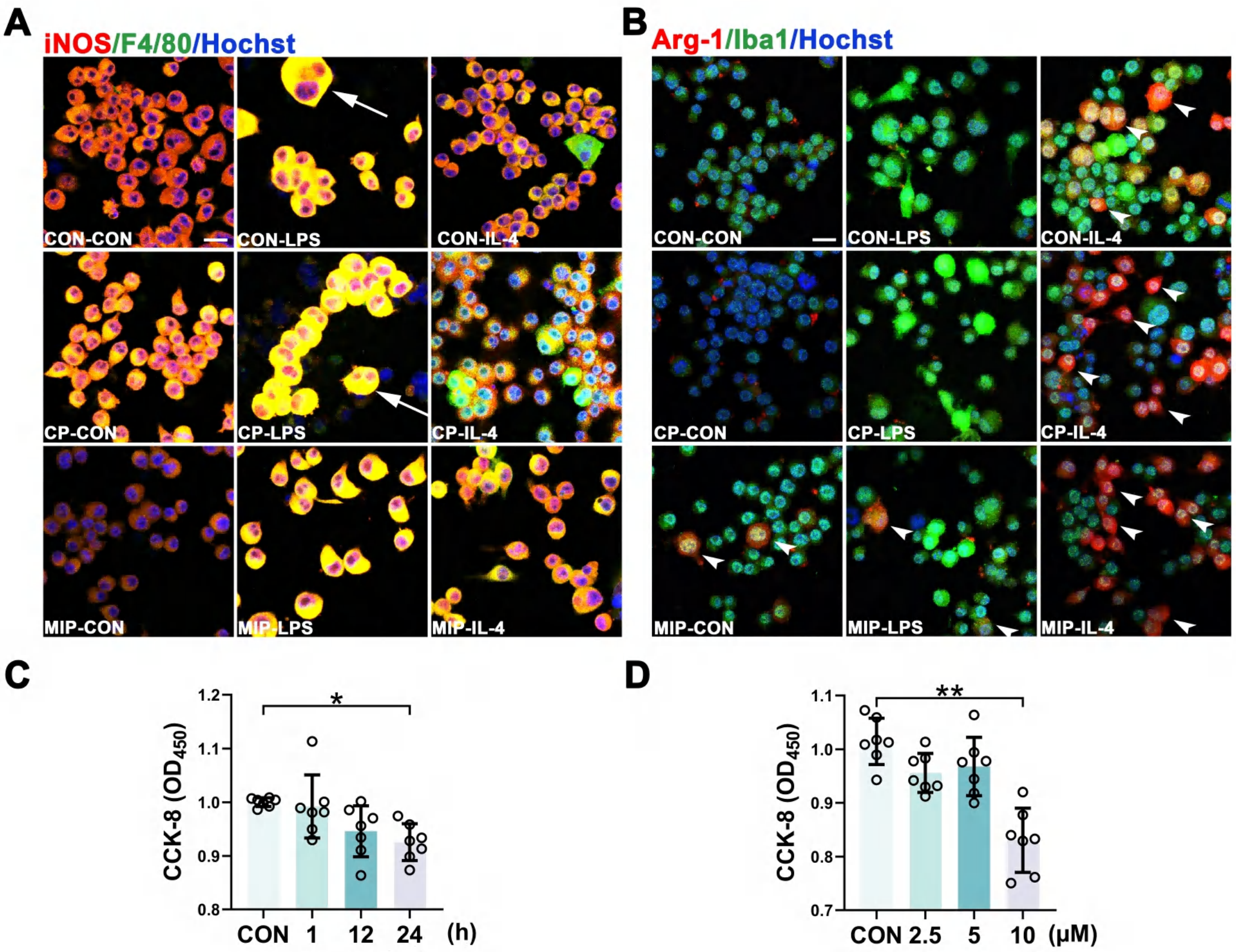

Figure S1. MyD88 inhibitory peptide attenuates LPS-induced iNOS and increases Arg-1^{+} cells in N9 microglia. (A) Representative N9 microglia stained for iNOS (red) and F4/80 (green); Hoechst (blue). (B) Representative N9 microglia stained for Arg-1 (red) and Iba1 (green); Hoechst (blue). (C) CCK-8 assay for microglia treated with ST2825 over different time periods (1, 12, and 24 h). (D) CCK-8 assay for microglia treated with different concentrations of ST2825 (2.5, 5, and 10 μM). Data are mean ± SD; one-way ANOVA with Tukey's post hoc test. Scale bars: 20 μm. Statistical significance: *$p < 0.05$, **$p < 0.01$.

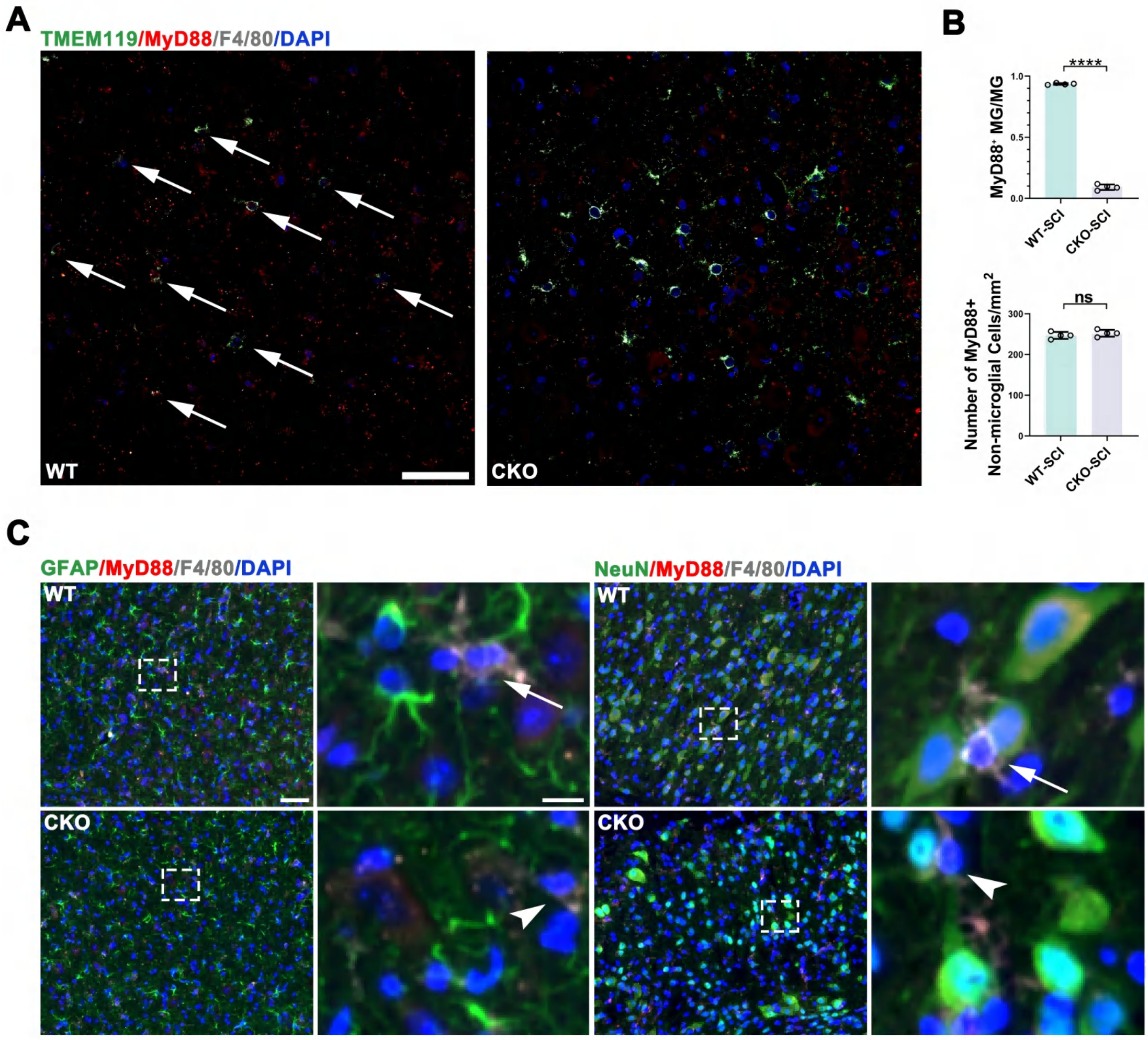

Figure S2. Validation of microglia-specific MyD88 deletion in cKO mice. (A) Representative sections of uninjured spinal cords stained for TMEM119 (green), MyD88 (red), F4/80 (white), and DAPI (blue). Arrows indicate the colocalization of microglial markers with MyD88 in WT mice, which is largely lost in cKO mice. (B) Quantification of MyD88$^+$TMEM119$^+$F4/80$^+$ cells and MyD88$^+$TMEM119$^-$F4/80$^-$ cells in spinal cord. (C) Representative images of spinal cords stained for MyD88 (red) and F4/80 (white) along with GFAP (green) or NeuN (green). Arrows indicate MyD88 colocalization within F4/80$^+$ cells. Arrowheads indicate F4/80$^+$ cells in the cKO-SCI group lacking MyD88 expression. Data are mean ± SD; n = 4 mice/group; unpaired two-tailed *t*-test. Scale bars: 50 µm (overview), 10 µm (single-cell views). Statistical

significance: ****$p < 0.0001$; ns, not significant.

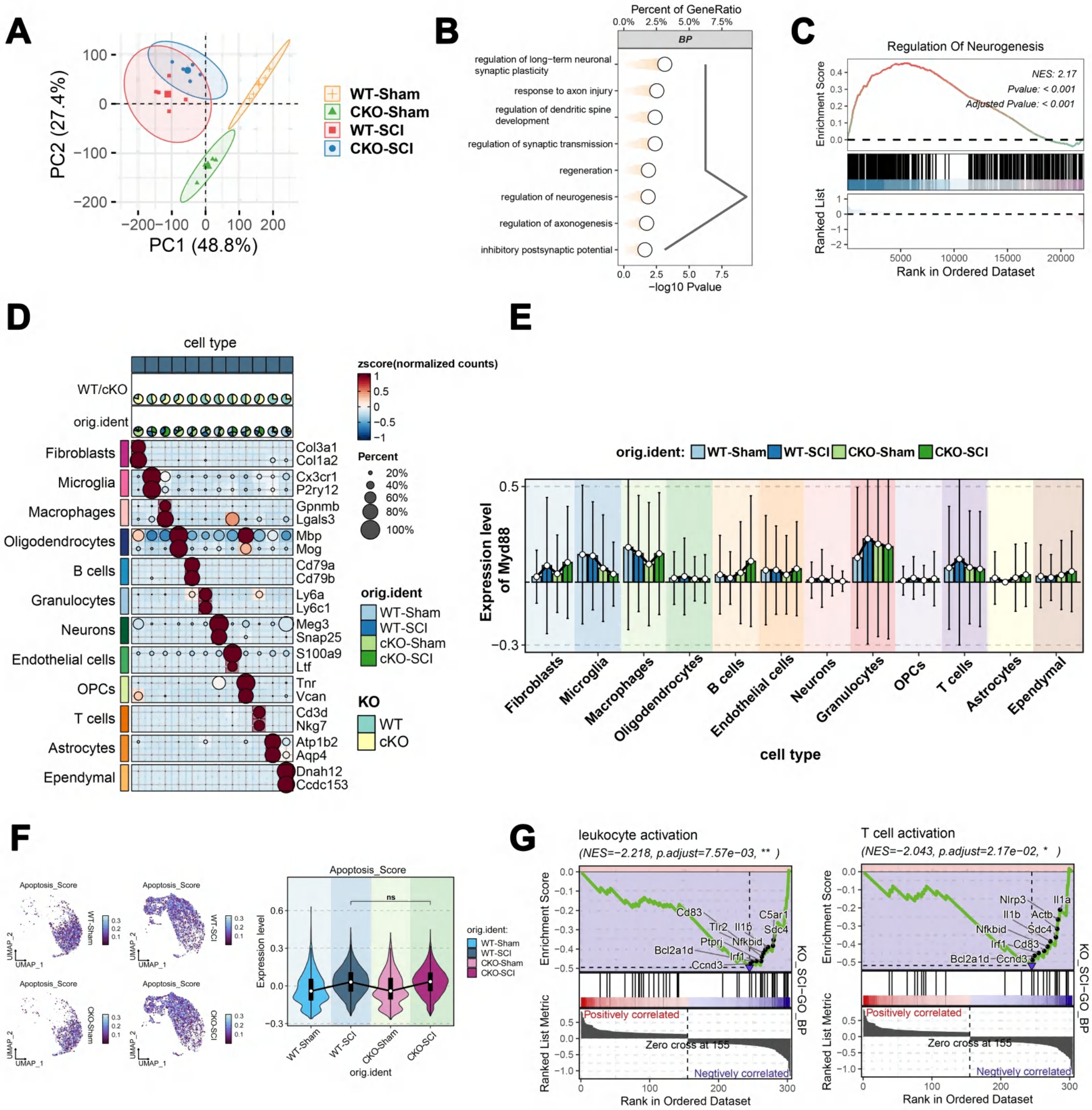

Figure S3. Bulk and single-cell transcriptomic analyses after microglial MyD88 deletion. (A) PCA of bulk RNA-seq profiles from sham and 3 dpi spinal cords. (B) GO terms enriched among upregulated genes in cKO-SCI versus WT-SCI. (C) GSEA plot for “regulation of neurogenesis” in cKO-SCI. (D) Dot plot of canonical markers used to annotate major cell populations in scRNA-seq. (E) MyD88 expression across major cell types in scRNA-seq across groups. (F) UMAP feature plots and violin plots showing the distribution and quantification of the apoptosis score across experimental groups. (G) GSEA plot showing lower enrichment of immune activation programs in

cKO-SCI microglia.

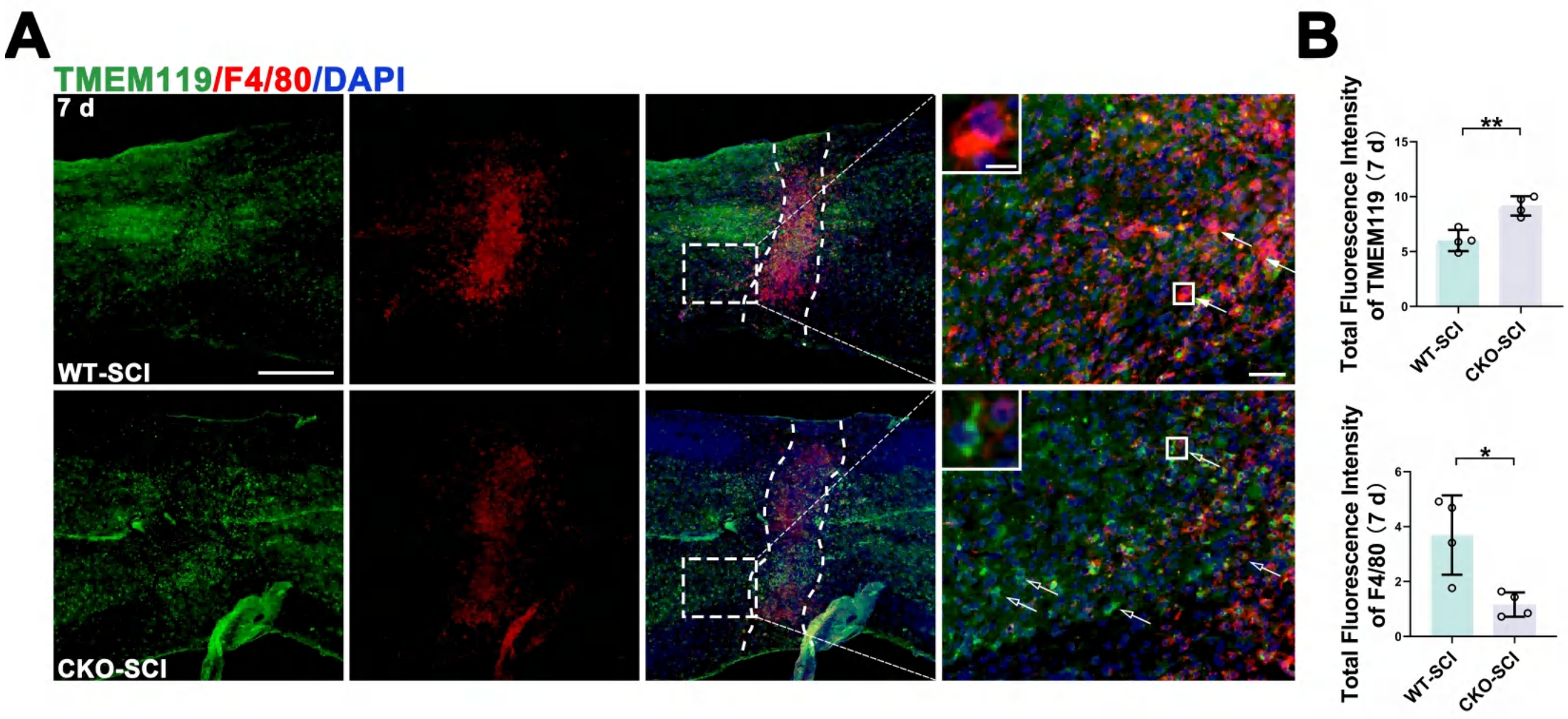

Figure S4. Accelerated recovery of microglia homeostasis in cKO mice after SCI. (A) Representative longitudinal sections at 7 dpi stained for TMEM119 (green) and F4/80 (red); DAPI (blue). (B) Quantification of TMEM119 and F4/80 fluorescence intensity at 7 dpi. Data are mean ± SD; n = 4 mice/group; unpaired two-tailed *t*-test. Scale bars: 500 μm (overview), 50 μm (insets), 10 μm (single-cell views). Statistical significance: *$p < 0.05$, **$p < 0.01$.

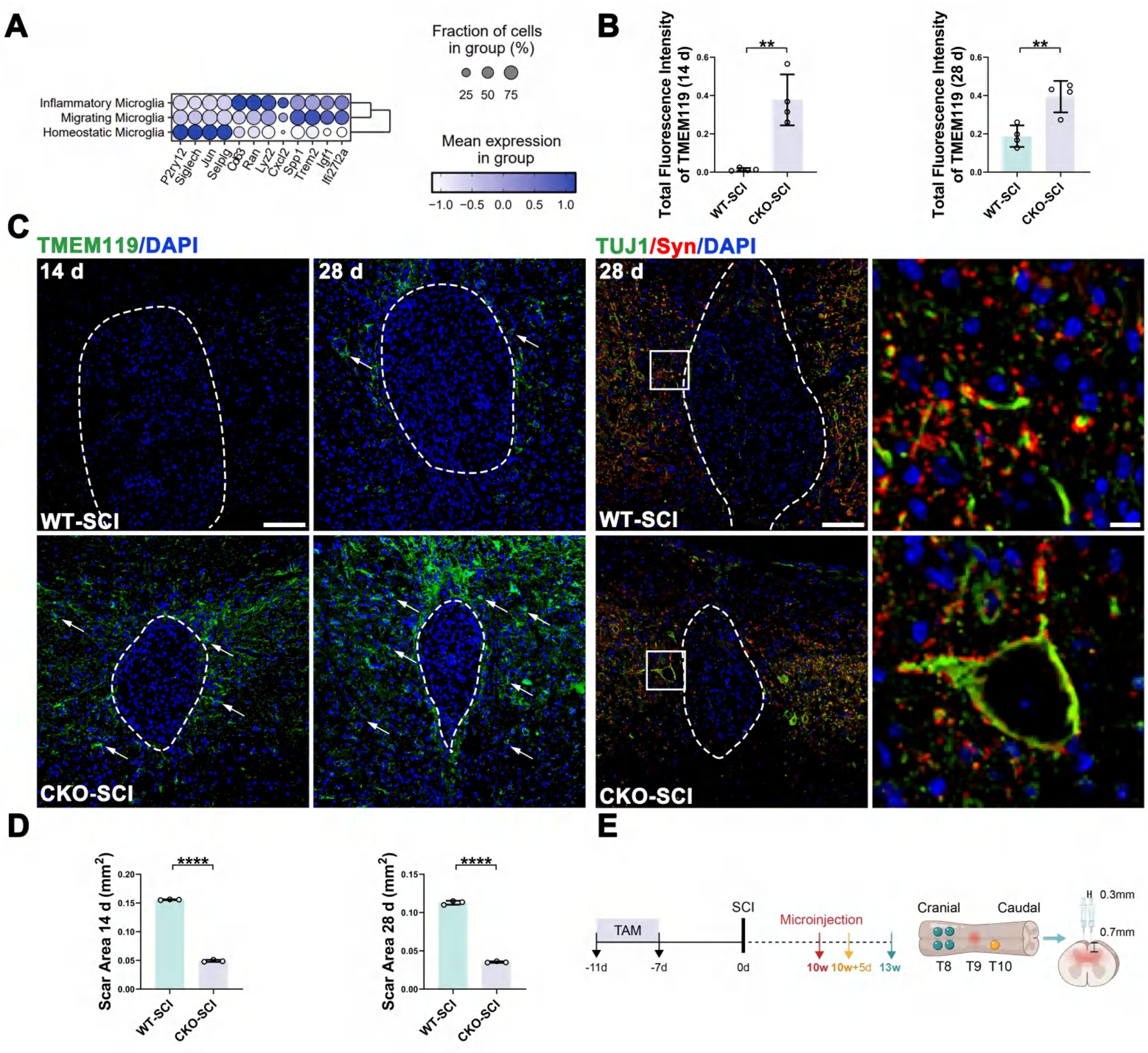

Figure S5. MyD88 cKO promotes microglial homeostasis and tissue repair after SCI. (A) Dot plot of representative marker genes across homeostatic, migrating, and inflammatory microglial states. Dot size indicates the fraction of expressing cells; color intensity indicates scaled average expression. (B-D) Sustained TMEM119 signal and reduced scar area in microglial MyD88 cKO mice. Data are mean ± SD; n = 4 mice/group; unpaired two-tailed *t*-test. Scale bars: 100 μm (overview), 10 μm (insets). Statistical significance: $**p < 0.01$, $****p < 0.0001$. (E) Schematic of tamoxifen induction, SCI, tracer delivery, and tissue collection. rAAV9-hSyn-EGFP was injected cranial to the lesion (four sites), and Fluoro-Gold was injected caudal to the lesion (one site). Coordinates relative to the dorsal surface are indicated (0.3 mm lateral, 0.7

mm depth).

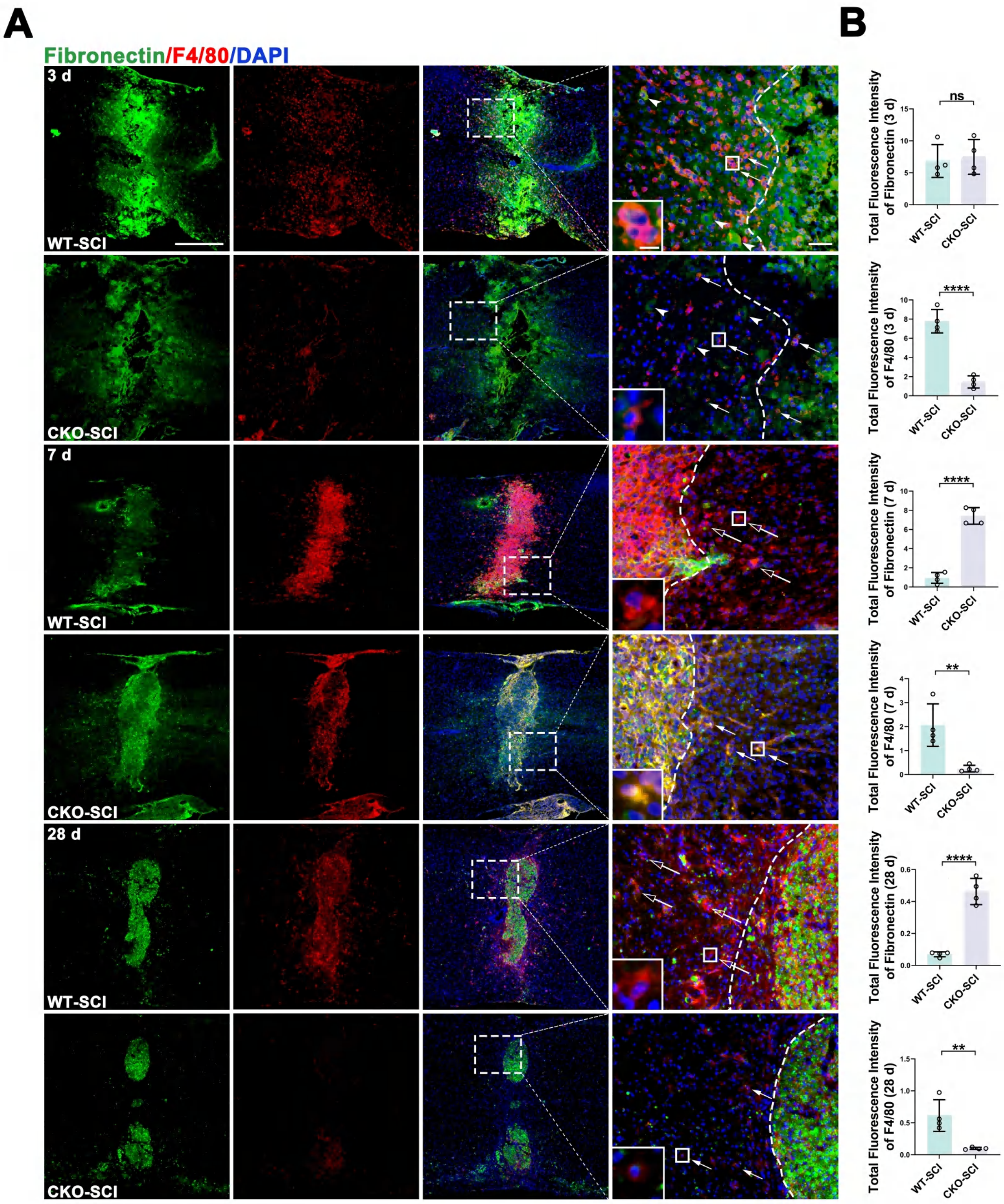

Figure S6. Fibronectin reveals sustained perilesional expression in cKO-SCI microglia compared with WT-SCI. (A) Representative longitudinal sections at 3, 7, and 28 dpi stained for fibronectin (green) and F4/80 (red); DAPI (blue). (B) Quantification of total fluorescence intensity of fibronectin and F4/80 at 3, 7, and 28 dpi. Data are mean ± SD; n = 4 mice/group; unpaired two-tailed *t*-test. Scale bars: 500 μm (overview), 50 μm (insets), 10 μm (single-cell views). Statistical significance: ***p*

< 0.01, ****$p < 0.0001$; ns, not significant.

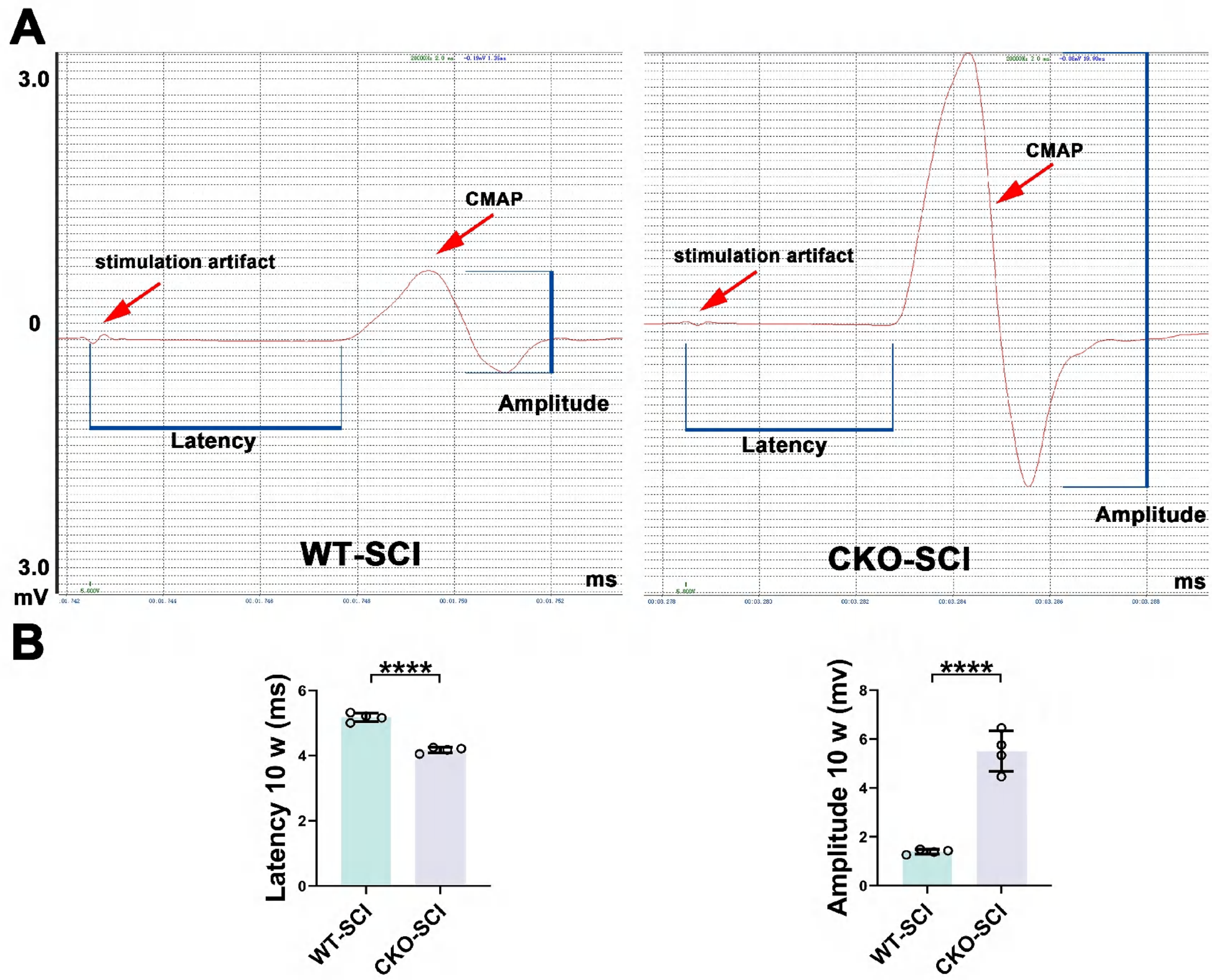

Figure S7. cKO-SCI mice exhibit improved electrophysiological function compared with WT-SCI controls. (A) Representative CMAP recordings from WT-SCI and CKO-SCI mice. (B) Quantification of CMAP latency and amplitude at 10 weeks post-injury. Data are mean ± SD; n = 4 mice/group; unpaired two-tailed *t*-test. Statistical significance: $****p < 0.0001$.

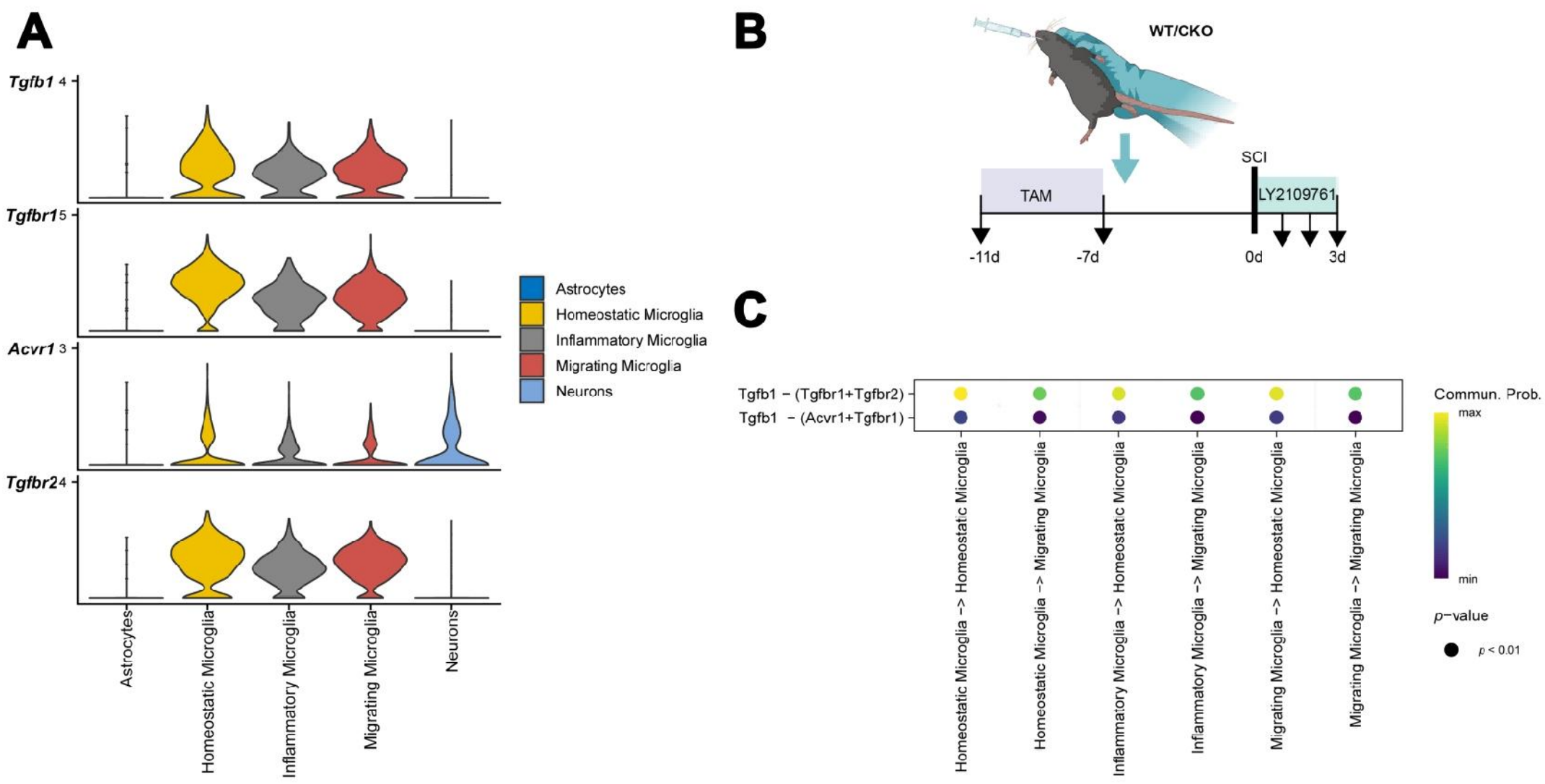

Figure S8. (A) Violin plots showing the expression levels of *Tgfb1* and its receptors (*Tgfbr1*, *Tgfbr2*, *Acvr1*) across major cell types. (B) Schematic diagram illustrating the experimental design for SCI and LY2109761 treatment. (C) Dot plot showing the communication probabilities of *Tgfb1*-*Tgfbr1*/*Tgfbr2* and *Tgfb1*-*Acvr1*/*Tgfbr1* ligand-receptor interactions among microglial subpopulations.

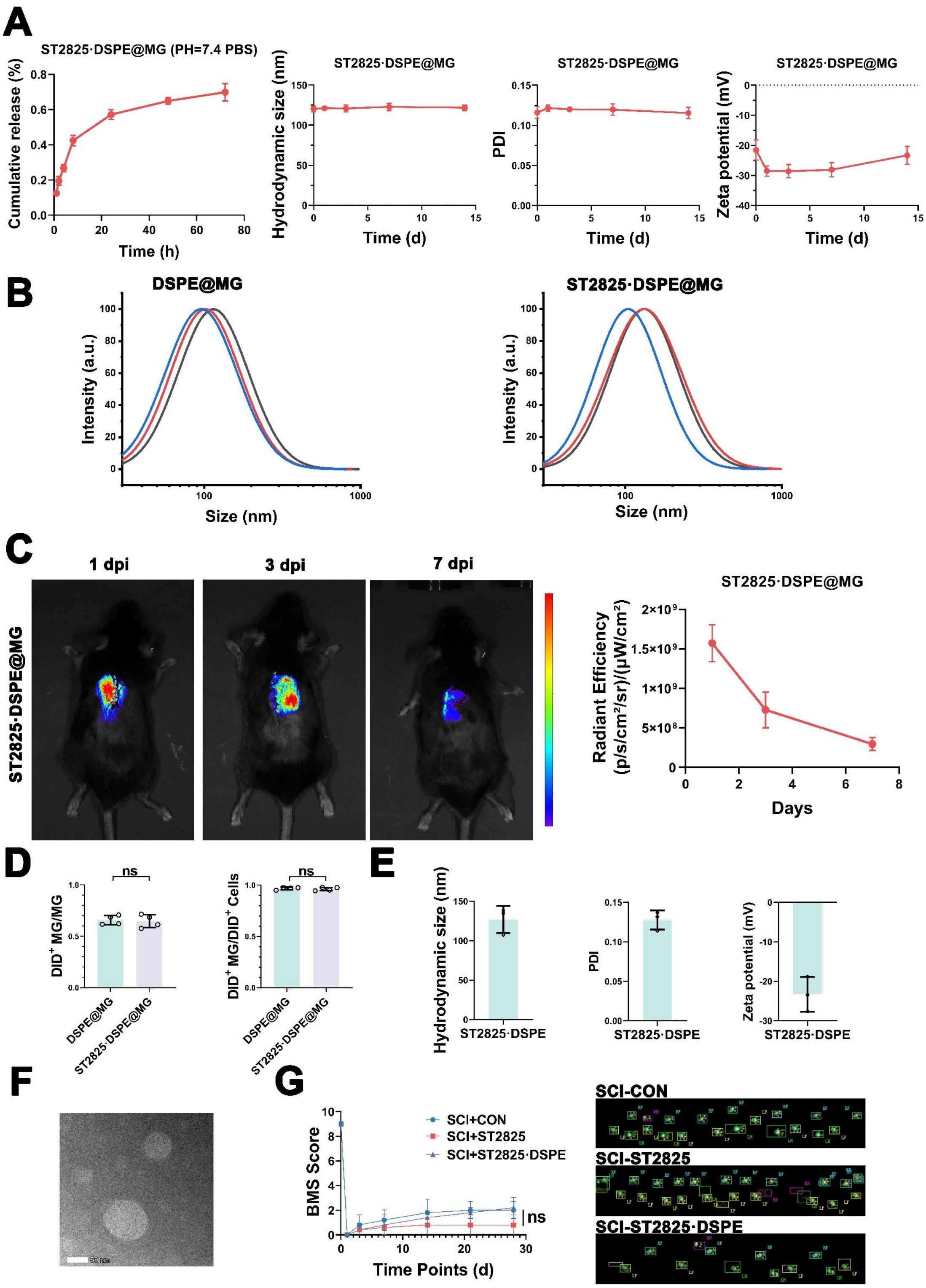

Figure S9. (A) *In vitro* drug release assays and colloidal stability tests of ST2825·DSPE@MG. (B) Size distribution of DSPE@MG and ST2825·DSPE@MG measured by DLS. (C) *In vivo* fluorescence imaging of DiD-labeled nanoparticles from 1 to 7 days post-administration, with quantification of radiant efficiency. (D)

Quantification of the DiD$^+$ cells in the perilesional region at 3 dpi. (E) DLS characterization of plain liposomes loaded with ST2825. (F) Representative TEM image of plain liposomes loaded with ST2825. Scale bars: 50 nm. (G) BMS scores and footprint analysis of the indicated groups. Data are mean ± SD; for (A, B, E), n = 3; for (C, D), n = 4 mice/group (unpaired two-tailed t-test for D). for (G), n = 5 mice/group (two-way repeated-measures ANOVA); ns, not significant.

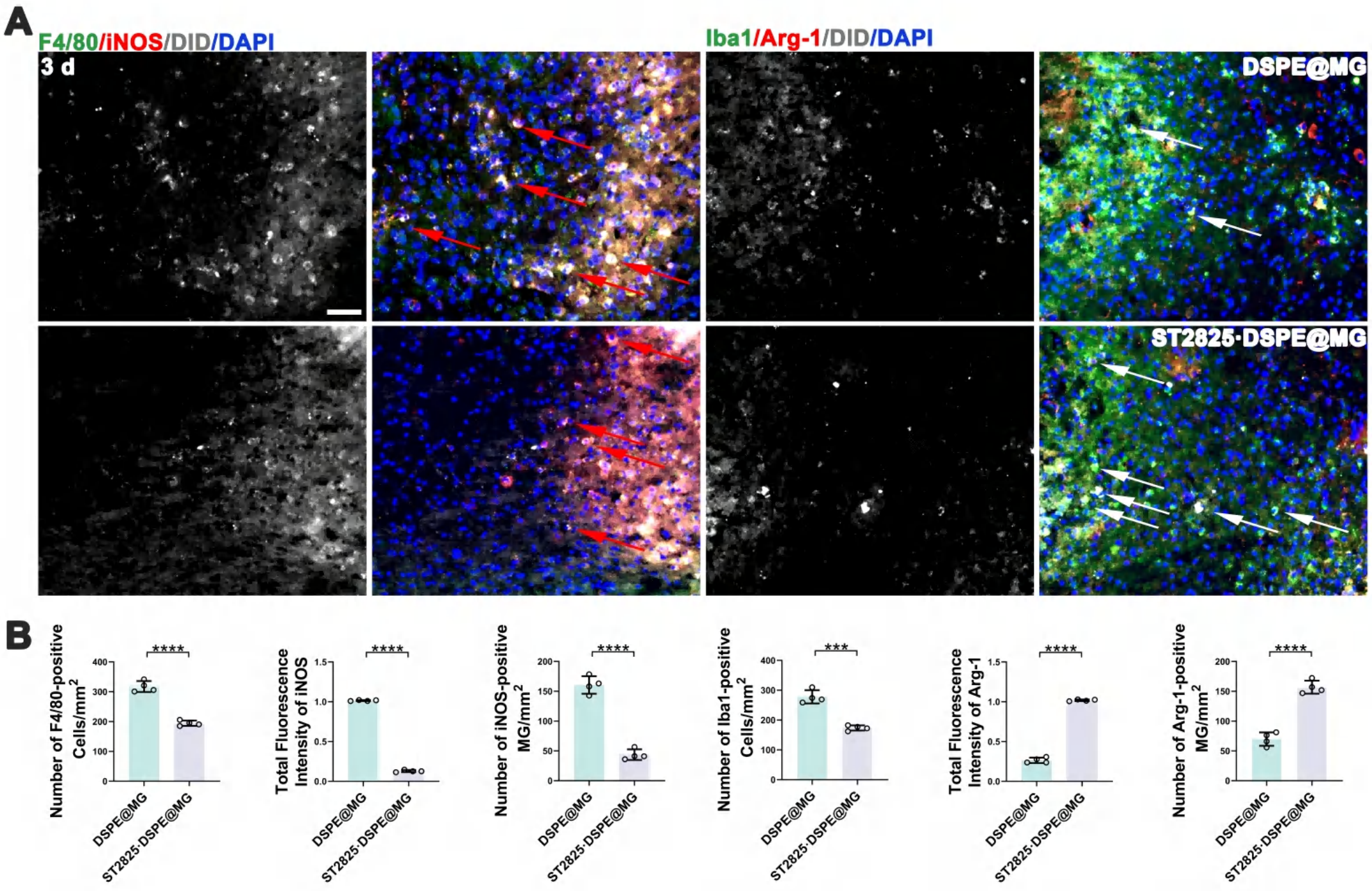

Figure S10. (A) Representative sections in the lesion area of male mice at 3 dpi stained for F4/80 (green) and iNOS (red), and Iba1 (green) and Arg-1 (red); DiD (white); DAPI (blue). (B) Quantification of lesion-region microglia and markers at 3 dpi. Data are mean ± SD; n = 4 mice/group; unpaired two-tailed *t*-test. Scale bar, 50 µm. Statistical significance: *** $p < 0.001$, **** $p < 0.0001$; ns, not significant.
